# Reference Intervals of Central Aortic Blood Pressure and Augmentation Index Assessed with an Oscillometric Device in Healthy Children, Adolescents, and Young Adults from Argentina

**DOI:** 10.1155/2018/1469651

**Published:** 2018-04-23

**Authors:** Alejandro Diaz, Yanina Zócalo, Daniel Bia, Edmundo Cabrera Fischer

**Affiliations:** ^1^Instituto de Investigación en Ciencias de la Salud, UNICEN, CONICET, Tandil, Argentina; ^2^Physiology Department, School of Medicine, Centro Universitario de Investigación, Innovación y Diagnóstico Arterial (CUiiDARTE), Republic University, General Flores 2125, 11800 Montevideo, Uruguay; ^3^Instituto de Medicina Traslacional, Trasplante y Bioingeniería (IMTTyB), Universidad Favaloro, CONICET, Buenos Aires, Argentina

## Abstract

Age-related reference intervals (RIs) of central (aortic) systolic blood pressure (cSBP) and augmentation index (cAIx) obtained from large healthy population are lacking in Argentina (South America).* Aims.* To analyze the existence of associations among cSBP and cAIx with demographic, anthropometric, and hemodynamic parameters and to generate percentile curves and RIs adjusted to each level of age and gender and/or body height. cSBP and cAIx were measured in 1038 healthy children, adolescents, and young adults. First, we evaluated if RIs for males and females were necessary using correlation and covariate analysis. Second, mean (M) and standard deviation (SD) age-related equations were obtained for cSBP and cAIx, using parametric regression methods based on fractional polynomials. Third, age specific percentiles curves were generated. Fourth, body height specific percentiles curves were generated using a similar procedure. The obtained equations (considering age as independent variable) for all subjects (cSBP^0.26^ and (cAIx + 12.001)^0.5^) were as follows: cSBP Mean = 3.0581 + 0.2189 log(Age) − 0.001044Age; cSBP SD = −0.03919 + 0.1535 log(Age) − 0.004564Age; cAIx mean = 9.5226 − 6.1599 log(Age) + 0.1450Age; cAIx SD = 1.3880 − 0.8468 log(Age) + 0.03212Age. This study, performed in Argentinean healthy children, adolescents, and young adults with ages of 5 to 22 years, provides the first RIs and percentile curves of cSBP and cAIx. Additionally, specific body height-related cAIx percentiles are reported for the analyzed population. The RIs and percentiles contribute to the knowledge of arterial dynamic evolution along the normal aging process and the interpretation of data obtained in clinical research and daily clinical practice.

## 1. Introduction

The analysis and monitoring of vessels morphology and function in young people have become of major interest for both pediatricians and adult specialists in order to preserve and optimize cardiovascular health from childhood to adulthood [1]. The noninvasive assessment of arterial stiffness (AS) provides valuable information of structural and functional state of the vascular system, beyond that of the conventional blood pressure (BP) measurements [2]. For instance, increases in AS in adolescents and young adults are associated with left ventricular mass index, independently of traditional cardiovascular risk factors (CRFs) [3]. Screening for AS may be useful to identify subjects with cardiovascular risk in children, adolescents, and young adults' populations [3].

There are several parameters to assess AS being frequently used the aortic pulse wave velocity (PWV) and augmentation index (AIx) of the aorta and the brachial artery. PWV and AIx increase with the arterial aging process [4–6]. Is important to take into account the fact that PWV is widely considered a direct marker of AS [7], while the AIx is a well-known modality to express arterial wave reflections, often considered as a surrogate index of AS [6–8].

Linked to AIx assessment, central (aortic) systolic blood pressure (cSBP) is a parameter that strongly reflects vascular changes of central elastic arteries compared to peripheral (brachial) BP, both in adults and children and adolescents [9, 10]. Moreover, changes in cSBP levels affect the heart, brain, and kidneys which are directly exposed to them [11].

Central arterial and haemodynamic parameters (cAIx and cSBP) can be estimated noninvasively using a good number of techniques. They include Doppler ultrasound, applanation tonometry, oscillometry, and magnetic resonance imaging; all of them are associated with its own strengths and weaknesses [2, 12]. Oscillometric devices have been suggested to be especially adequate in children and adolescents, since the methodology includes the use of a simple pressure cuff and have the advantages of being relatively fast, operator independent and require minimal cooperation of the patient [13, 14].

Independently of the technique or device used to assess the arterial properties, there are several gaps in the approach of cSBP and cAIx measurements [1], performed in pediatric clinical practice such as (a) what are the interval references (RIs) of cSBP and cAIx values for each measurement? And (b) do they differ by gender and/or body height?

The 2016 European Society of Hypertension guidelines for the management of high BP in children and adolescents do not recommend the routine assessment of arterial parameters and cSBP, however, highlights the need to increase knowledge in the field of arterial parameters as markers of function of the vascular system [15]. Furthermore, the 2013 ESH/ESC guidelines for the management of arterial hypertension considered that cSBP can provide useful information in young patients with isolated systolic hypertension [16]. Moreover, cSBP may be especially relevant in asymptomatic children and adolescents incidentally found to have isolated systolic hypertension without target organ damage [15].

Unfortunately, at present few epidemiological studies in healthy pediatric populations are available, in which RIs of cSBP and cAIx were obtained using brachial oscillometric cuff method [17, 18]. To the best of our knowledge, the largest databases of RIs of cSBP and cAIx in pediatrics come from Hungary [17] and Germany, in which the Arteriograph and Mobil-O-Graph System were respectively used [18].

Considering the lack of knowledge in terms of cSBP and cAIx reference values for children, adolescents, and young adults from Argentina, our research purposes were as follows:

(1) to analyze the existence of associations among cSBP and cAIx with demographic, anthropometric, and hemodynamic parameters,

(2) to generate percentile curves and RIs adjusted to each level of age and (if required) gender and/or body height.

## 2. Materials and Methods

This study is part of a project started in 2014 in Tandil city (Buenos Aires Province), Argentina, aimed at investigating the prevalence of CRFs. Preliminary data obtained in this location have been previously reported [19–21]. Tandil is located 360 Km south of Buenos Aires City (37°19′08′′S-9°08′05′′W).

The study protocol was approved by the Institutional Ethics Committee and was carried on in agreement with the Declaration of Helsinki and the Good Clinical Practice Guidelines. Written informed consent was obtained from the participants or authorized person.

Asymptomatic children, adolescents, and young adults (5–22 years old) from the Tandil community were considered for enrolment in this study. Subjects were submitted to clinical interview, blood sampling evaluation, and anthropometric assessment, carried out in all cases by the same group of physicians. Blood samples were obtained after 9–12 hours of fasting. Glycaemia, lipid profile, and kidney functional parameters were determined. Anthropometric evaluation and a brief clinical interview allowed assessing CRFs exposure. Asymptomatic subjects included in the study met the following criteria: (1) none had history of cardiovascular, pulmonary, or renal disease; (2) normal peripheral BP at the time of examination (BP ≤ 140/90 mmHg in adults and BP < 90th percentile in subjects < 16 years old) [15]; (3) none were taking medications (antihypertensive, antihyperlipidemic, or antidiabetic drugs); (4) all included subjects had glycaemia < 6.11 mmol/L (<110 mg/dl), total blood cholesterol levels < 5.17 mmol/L (<200 mg/dl) [16], and normal serum triglycerides levels < 1.69 mmol/L (<150 mg/dl), ≤1.5 mmol/L (<130 mg/dl), and ≤1.13 mmol/L (<100 mg/dl) for subjects older than 18 years, subjects between 10 to 17 years, and children under 10 years, respectively [15, 22].

Current and past smokers, diabetic, obese subjects (body mass index [BMI] ≥ 30 kg/m^2^ for adults or BMI ≥ 97th percentile for subjects under 18 years old), hypertensive subjects, or subjects with averaged high brachial BP levels at the time of the study were excluded. To this end, peripheral (brachial) BP measurements were obtained using fully automatic sphygmomanometers, operating on oscillometric principle (705IT, Omron Healthcare Inc., USA). Adults' BP levels were classified following guidelines for the management of arterial hypertension [16]. Thus, hypertension was defined as peripheral systolic BP (pSBP) ≥ 140 mmHg and/or peripheral diastolic BP (pDBP) ≥ 90 mmHg. In turn, brachial BP levels in children and adolescents were categorized, taking into account sex, age, and body height, according to criteria of the American Pediatrics Association and the European Society of Hypertension report [15].

### 2.1. Augmentation Index and Blood Pressure Measurements

All measurements were performed using the Arteriogrph system (Arteriograph; TensioMed Ltd, Budapest, Hungary) after 10 minutes of rest in a quiet room with stable temperature (22 ± 1°C). The Arteriograph system is an operator-independent noninvasive device that applies an oscillometric, occlusive technique by use of an upper-arm cuff to register brachial pressure waves.

The mentioned device was used to assess simultaneously central (aortic) systolic and pulse pressure (cSBP, cPP) and peripheral (brachial) systolic, diastolic, pulse, and mean BP levels (pSBP, pDBP, pPP and MAP, resp.). The measurement procedure takes approximately 3 minutes and is operatively comparable to an automatic digital BP oscillometric measurement. In this study, the same device was used for all measurements. The working principle and the invasive validations of this method have been detailed previously [6, 23]. The method is based on the physiological fact that the early (*P*1) systolic pulse pressure wave of the aorta, generated by the left ventricle ejection, travels along the aorta and is reflected (*P*2) from the area of the aortic bifurcation. Occluding the brachial artery by pressurizing the cuff 35–40 mmHg above the actual SBP creates easily distinguished, pronounced pressure peaks in the cuff. Separated in this way, early and late systolic waves can be recorded. The time lapses between the peaks of *P*1 and *P*2 are equal to the travel time of the aortic pressure wave from the aortic root to the bifurcation and back. By halving this time and measuring the sternal notch, pubic bone distance (which is rather close to the true aortic length [24], the Ao-PWV can be calculated (Ao-PWV = jugulum-symphysis distance/transit time). Additionally, the Arteriography system calculates the brachial augmentation index (bAIx) using the formula: bAIx (%) = (*P*2 − *P*1/PP) × 100. The cAIx was calculated based on invasive measurements previously reported [23], which showed a very strong linear correlation between bAIx and cAIx (*R* = 0.94, *p* < 0.001). These studies showed a strong and significant correlation between the invasively obtained values and oscillometrically measured cAIx, cSBP, and aortic PWV. Taking into account the influence of heart rate on cAIx [25], in each subject the mentioned index was calculated corresponding to 75 beats by minute (cAIx-HR75).

### 2.2. Data Analysis

In this research, a step-wise data analysis was done as is described in the following.

*First*, aiming at determining whether RIs analysis for cAIx and cAIx-HR75 was necessaries, we separately assessed the degree of association (correlation) and equivalence (agreement) between cAIx and cAIx-HR75 levels, by studying potential mean and/or proportional differences (errors) between measurements and constructing limits of agreement (correlation and Bland-Altman analysis). As a first result, specific RIs for cAIx and cAIx-HR75 were defined as not necessaries. About this, both variables showed a great association (cAIx = −0.2753 + 0.9957 · cAIx-HR75; *R* ^2^ = 0.9953). However, despite a statistically significant difference between both variables (mean error = −0.3167%, *p* < 0.0001; mean error 95% confidence interval = −0.3522% to −0.2811%; mean error standard deviation (SD) = 0.5836%, 95% confidence interval = −1.4605% to 0.8272%), the difference was not considered clinically significant, as to force the generation of RIs separately (for cAIx and cAIx-HR75).

Second, we evaluated if RIs for males and females were necessary. To this end, bivariate and point-biserial correlations were done (Table 2), and after that gender influence was examined before and after adjustment for cofactors (i.e., age, BP) applying covariance analysis (ANCOVA) (Table 3). ANCOVA allows comparing one variable (i.e., cSBP or cAIx) in two or more groups (i.e., males versus females) taking into account (or to correct for) variability of other variables, called covariates or cofactors. With this purpose, correlations were done to identify demographic, anthropometric (i.e., body height, body weight) and/or hemodynamic (i.e., heart rate, pSBP, and pDBP) variables that in theory could be considered as cofactors in covariate analysis. Once the variables significantly associated with cSBP and cAIx were identified, ANCOVA analysis was done adjusting for them (Table 3). With this purpose, several models (cofactors combinations) were analyzed. Always, prior to the ANCOVA test, Levene's test for equality of variances and homogeneity of regression slopes test were performed. If Levene's test is positive (*p* < 0.05) then the variances in the groups are different (the groups are not homogeneous), and therefore the assumptions for ANCOVA are not met. Additionally, the interpretation of ANCOVA and the associated adjusted (or marginal) means relies on the assumption of homogeneous regression slopes for the compared groups (i.e., males and females); if this assumption is not met (*p* < 0.05) the ANCOVA results are unreliable. After statistical analysis, as a result, generation of specific cSBP and cAIx RIs for males and females was considered as necessaries.

Third, the mean value and SD age-related equations (for males and females) were obtained for cSBP and cAIx. With this purpose, parametric regression methods based on fractional polynomials (FPs), as described by Royston and Wright [26] and previously used to generate RIs for arterial parameters in the European Arterial Stiffness Collaboration Group methodological strategy [27, 28], were implemented using MedCalc Software (MedCalc, Ostend, Belgium). Briefly, fitting FPs for age specific cSBP and cAIx mean value and SD regression curves were defined using iterative procedure (generalized least squares, GLS). The obtained results enabled estimating age specific mean and SD for both parameters (cSBP and cAIx). For instance, cAIx mean equation could be cAIx = *a* + *b∗*age^*p*^ + *c∗*age^*q*^ + ⋯, where *a*, *b*, *c*,… are the coefficients, and *p*, *q*,… are the powers, with numbers selected from the set [−2, −1, −0.5, 0, 0.5, 1, 2, 3] estimated from the regression for the mean cAIx curve and likewise from the regression for the SD curve. Continuing the example, FPs with powers [1,2], that is, with *p* = 1 and *q* = 2, illustrate an equation with the form *a* + *b∗* age + *c∗*age^2^ [26]. The residuals were used to assess the model fit, which was deemed appropriate if the scores were normally distributed, with a mean of 0 and a SD of 1, randomly scattered above and below 0 when plotted against age. The best fitted curves, considering visual and mathematical criteria (Kurtosis and Skewness coefficients), were selected. Then, using the equations obtained for mean and SD, age specific percentiles were defined using the standard normal distribution (*Z*) (Tables 4, 5, and 6 for cSBP and Tables 7, 8, and 9 for cAIx). Age specific 1th, 2.5th, 5th, 10th, 25th, 50th, 75th, 90th, 95th, 97.5th, and 99th percentile curves were calculated as mean cAIx + Zp *∗* SD, where Zp assumed the values of −2.3263, −1.9599, −1.6448, −1.2815, −0.6755, 0, 0.6755, 1.2815, 1.6448, 1.9599, and 2.3263, respectively. The obtained equations were as follows:

(i) For all subjects (cSBP^0.26^ and (cAIx + 12.001)^0.5^),

(1) cSBP  Mean=3.0581+0.2189log⁡Age−0.001044AgecSBP  SD=−0.03919+0.1535log⁡Age−0.004564AgecAIx  Mean=9.5226−6.1599log⁡Age+0.1450AgecAIx  SD=1.3880−0.8468log⁡Age+0.03212Age.

(ii) For males (cSBP^1.13^ and (cAIx + 7.001)^0.53^),

(2) cSBP  Mean=146.7373+5.4105log⁡Age+1.7801AgecSBP  SD=−10.3739+39.2556log⁡Age−1.2532AgecAIx  Mean=7.4365−3.7152log⁡Age+0.05135AgecAIx  SD=−1.2276+3.4356log⁡Age−0.1113Age.

(iii) For females (cSBP^0.26^ and (cAIx + 12.001)^0.66^),

(3) cSBP  Mean=2.9920+0.3501log⁡Age−0.007039AgecSBP  SD=−0.07844+0.2039log⁡Age−0.005824AgecAIx  Mean=21.8535−18.4253log⁡Age+0.5115AgecAIx  SD=3.2466−1.6274log⁡Age+0.03122Age.

Finally, a similar procedure was performed to obtain cAIx RIs considering the body height (BH) [17]. The obtained equations were as follows:

For males (cAIx + 7.001)^0.53^,

(4) cAIx  Mean=−16.3756+13.9683log⁡BH−0.06468BHcAIx  SD=−14.8123+8.6667log⁡BH−0.02016BH.

For females (cAIx + 12.001)^0.66^,

(5) cAIx  Mean=−46.6729+35.8589log⁡BH−0.1523BHcAIx  SD=38.0202−21.0915log⁡BH+0.06424BH.

In the equations detailed above, always cSBP, cAIx, Age, and BH were expressed in mmHg, %, years, and centimeters, respectively.

Limit of statistical signification was considered when a *p* value < 0.05 was found. Calculus of the minimum sample size required was calculated taken into account a normal distribution of the covariate (age) in the sample (in a conservative way) and a 95% and 90% limit of reference and confidence interval (two sided), respectively, with a 95% and 10% reference range and relative margin of error, respectively. In this research, the minimum required sample size for RIs construction (i.e., for males or females) was 377 subjects [29].

## 3. Results

### 3.1. General Characteristics of the Analyzed Population

In this research 1038 healthy subjects (576 males and 462 females) were included. In Table 1 the characteristics of all children, adolescents, and young adults are summarized, discriminating values obtained for males and females. Mean age for the whole population was 15.35 ± 3.15 years (range 5.00–21.92 y.o.) and no significant age differences were found (Table 1). In this cohort, males showed the highest body weight, body height, and BMI values (*p* < 0.001). Also, males exhibit significant higher jugulum-symphysis distance than females (*p* < 0.001). Mean values of pSBP and pPP obtained in males were significantly higher than those obtained in females (*p* < 0.001); on the contrary pDBP and HR were significantly lower in males compared with females. No significant differences related to gender in terms of pMAP values were observed. Moreover, males have higher mean values of cSBP and cPP than females (*p* < 0.05 and *p* < 0.001, resp.). See Table 1.

No significant gender differences in peripheral AIx, ejection duration, and Ao-PWV mean values were observed in the analyzed population. Obtained mean values of cAIx and cAIx-HR75 were always higher in females than in males (*p* < 0.001). See Table 1.

### 3.2. Analysis of cSBP and cAIx Associations

As was mentioned significant differences between cAIx and cAIx-HR75 were found; however absolutely values of these differences were really minimal (mean error = −0.3167, *p* < 0.0001; mean error 95% confidence interval = −0.3522 to −0.2811; mean error SD = 0.5836, 95% confidence interval = −1.4605 to 0.8272). Consequently, taking into account the RIs generated in terms of cAIx, it was evidenced that it was not necessary to generate RIs using cAIx-HR75 obtained values. That is to say, percentiles generated using cAIx or cAIx-HR75 were very similar and observed differences were lack of clinical relevance. In other words, the difference was not considered clinically significant, as to force the generation of RIs separately (for cAIx and cAIx-HR75).

Table 2 show the bivariate and point-biserial correlation study of cSBP, cAIx, and Age relationship with demographic, anthropometric, hemodynamic, and vascular parameters of the analyzed population. As can be seen in Table 2, the age and the anthropometric parameters show a positive and significantly association with cSBP and cAIx levels. Moreover, HR shows borderline statistic values of association with cSBP (*p* = 0.079).

Table 3 show a covariance (ANCOVA) study destined to determine if the gender discrimination would be necessary in the RIs of cSBP and cAIx generation using data collected in this research. Also in Table 3, the noncorrected and corrected marginals' means values with standard error and 95% confidence intervals are given for males and females. In the case of cSPB, a first analysis induces to think that adjustments using Models 1, 2, 3, and 4 show no statistically significantly gender differences. However, the comparison of regression lines obtained from males and females showed statistically significant differences; that is to say there are different slopes of the cSBP-age regression lines (see homogeneity of regression slope's test in Table 3(a)). As the ANCOVA analysis and the associated adjusted means values rely on the assumption of the homogeneity of regression slopes in the studied groups. If this assumption is not met (*p* < 0.05) the ANCOVA results are unreliable; consequently, in the analyzed population, it is absolutely necessary to perform different RIs for cSBP for males and females (Table 3(a)). Similar results were obtained when sex-related differences in cAIx were analyzed using Models 3 and 4 (Table 3(b)). Moreover, for all considered models, sex-related differences after Levene's test for equality of variances were also demonstrated (different in terms of the calculated variance). Again, if Levene's test is positive (*p* < 0.05) the variances in the analyzed groups should be considered statistically different (the groups are not homogeneous); so the assumption of homogeneity is not met and the ANCOVA analysis is invalid. As a consequence of the results obtained in cAIx ANCOVA analysis, sex-related RIs should be necessary in this population (Table 3(b)).

### 3.3. Reference Intervals (Percentile Analysis) Obtained in the Analyzed Population

**Figure 1:**
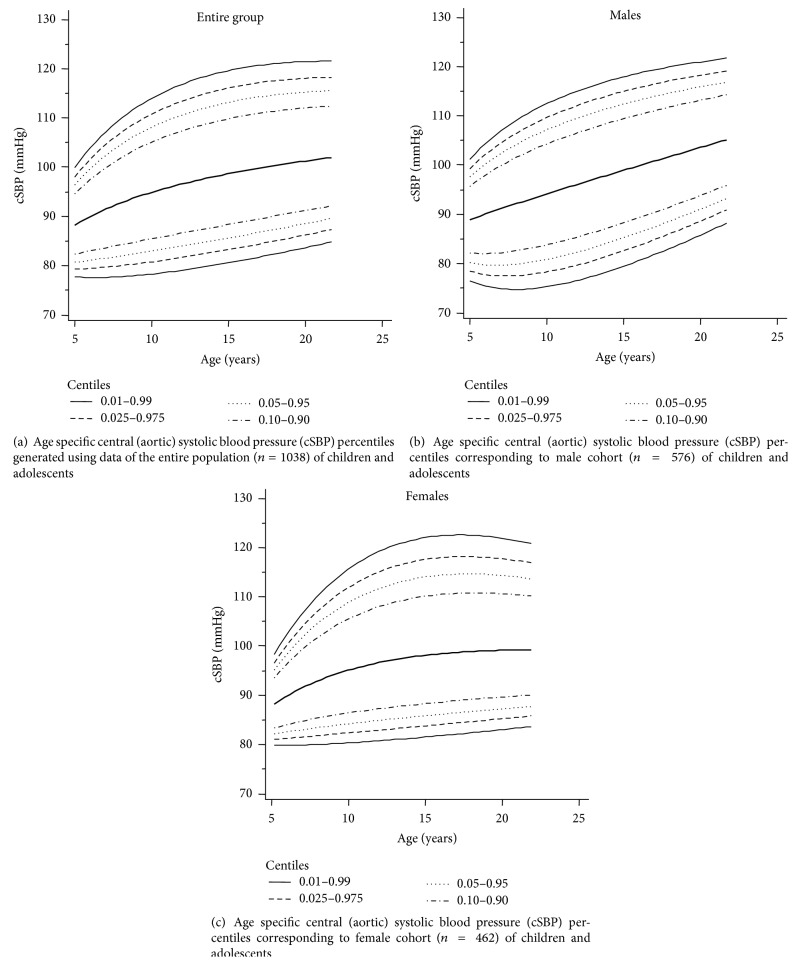


As is shown in Table 4, specific percentiles of age-cSBP values for five-year age RIs were generated including the entire population (*n* = 1038), that is, including males and females. Furthermore, a similar analysis was carried out using values corresponding to each year of age (Table A in Supplementary Materials). As seen in Table 5, age-cSBP percentiles corresponding to 5-year age intervals were generated for males (*n* = 576). A similar analysis was done for each year of age, as seen in Table B (see Supplementary Materials). Females (*n* = 462) were analyzed using a similar method, and the obtained RIs can be seen in Table 6 and Table C (see Supplementary Materials). Figures 1(a), 1(b), and 1(c) show specific age-cSBP percentiles for the entire population, males and females, respectively. As expected, there was a positive association between age and cSBP values, representing a gradual and continuous increase in terms of cSBP.

**Figure 2:**
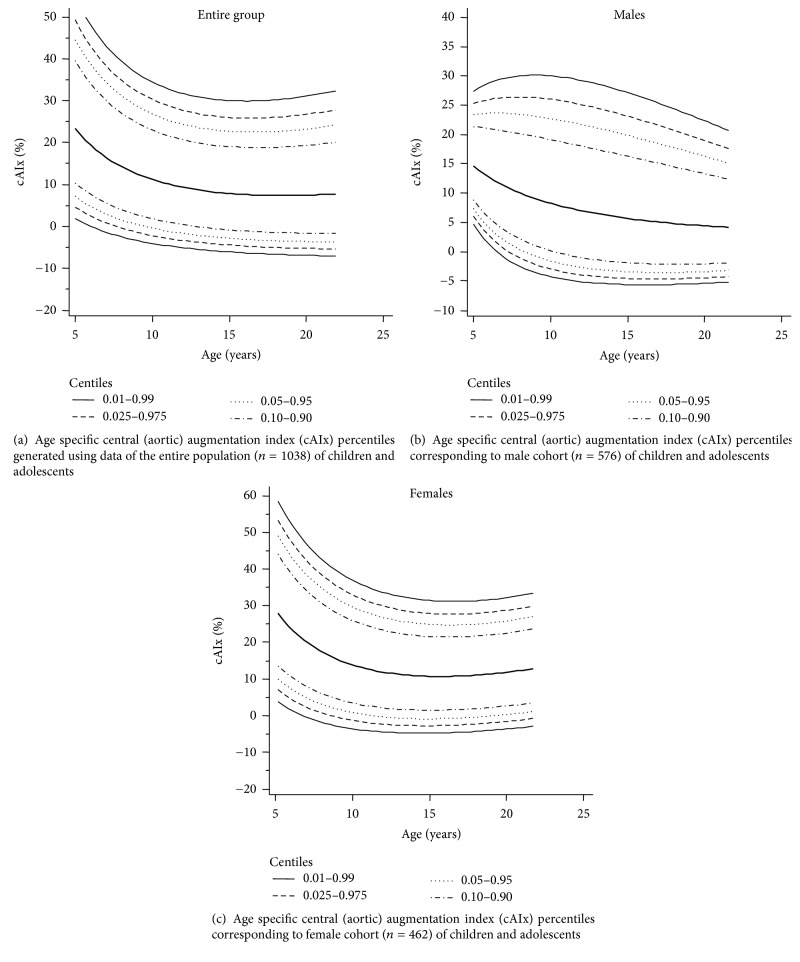


 Tables 7, 8, and 9 show specific percentiles of age-cAIx values for five-year age RIs corresponding to the entire population, males and females, respectively. Furthermore, a similar analysis was carried out using values obtained by each year of age (Tables D, E, and F in Supplementary Materials). Figures 2(a), 2(b), and 2(c) show specific age-cAIx percentiles for the entire population, males and females, respectively. As expected, there was a correlation between age and cAIx values.

**Figure 3:**
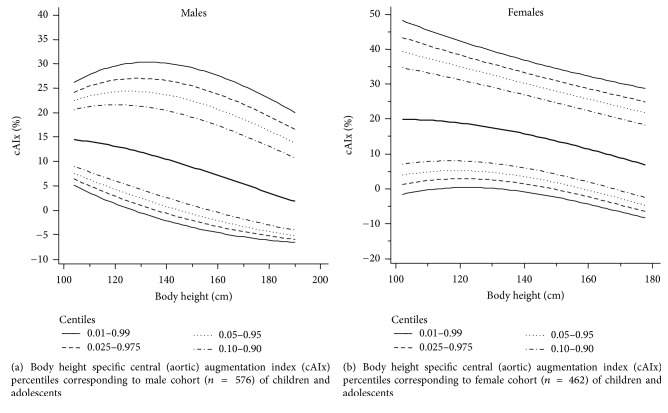


Specific percentile analysis of cAIx corresponding to each body height level (using 5 cm intervals) of the male cohort is shown in Table 10. Also in Table G, using 1 cm interval is provided in Supplementary Materials. Furthermore, in Table 11 and Table H of the Supplementary Materials, RIs corresponding to the female cohort are showed. Figures 3(a) and 3(b) show body height-cAIx percentiles, corresponding to males and females, respectively.

## 4. Discussion

This research provides the first Latin American database concerning RIs of cSBP and cAIx obtained on a large cohort of healthy children, adolescents, and young adults aged between 5 and 22 years. Moreover, the definition of RIs generated in this study will further improve our ability to identify young populations at high risk and represent a first step toward new approaches in the diagnosis and management of high BP states and/or other altered haemodynamic conditions found in childhood and adolescence. To the best of our knowledge, there are no widely accepted clear cutoff values for central aortic BP levels in pediatric clinical practice, in spite of recent efforts of European researchers [18]. In this context, our study provides percentiles for cSBP and cAIx representing a novel approach to optimize risk stratification in youths.

The cAIx inform about the arterial wave reflection component of the left ventricular afterload. Values of cAIx are mainly determined by two factors: (a) aortic stiffness (which in turn determines the “central to peripheral” and “peripheral to central” forward and backward wave components propagation velocities) and (b) distance between aortic root and major wave reflection sites (i.e., aortic bifurcation at the level of pelvic arteries and include small arteries) [30]. Increases in aortic stiffness enhance the speed of wave reflection (resulting in an earlier return of the reflected wave to the ascending aorta) and increase the magnitude of the backward or reflected wave (resulting in an increase in aortic pulse pressure and left ventricle afterload). As a result, frequently when the AS increases the higher the cAIx is [30]. Consequently, the cAIx inform about the vascular wall state as a surrogate index of AS [6, 23]. Additionally, reduced body height is associated with early arrival of reflected waves to the ascending aorta, and consequently is associated with increased cAIx. Furthermore, high cAIx values implicate that increases of cSBP and/or cPP are determined by early arrival of reflected waves (during left ventricular systolic period). In other words, jointly analyzing the cAIx and cSBP or cPP, it is possible to determine if the levels of cSBP or cPP (i.e., increased) are due (or not) to an increase in the amplitude or early arrival of the reflected waves. Taking into account the determinant of cAIx, subjects with similar levels of aortic stiffness could exhibit significant differences in cAIx associated with differences in body height. This is particularly important in children and adolescents in which both AS and body height are continuously changing. From this point of view, cAIx would be a very representative index of arterial dynamics and left ventricular afterload, since it evaluates simultaneously AS and changes associated with growth process.

On the other hand, levels of BP are mainly determined by cardiac and vascular factors. Higher (and faster) left ventricular stroke volume is associated with high levels of cSBP and cPP. Otherwise, AS and arrival time and amplitude of wave reflections are the main determinants of cSBP and cPP, while peripheral vascular resistances and cardiac output are the main determinants of MBP and cDBP. In this context, it is important to emphasize that simultaneous measurements of central (aortic) BP and cAIx (using a single device) in children and adolescents allow us not only to know if cSBP and cPP are within physiological levels, but also in case of abnormal values to realize if they were associated or not with increased wave reflection levels (cAIx) [31, 32].

**Figure 4:**
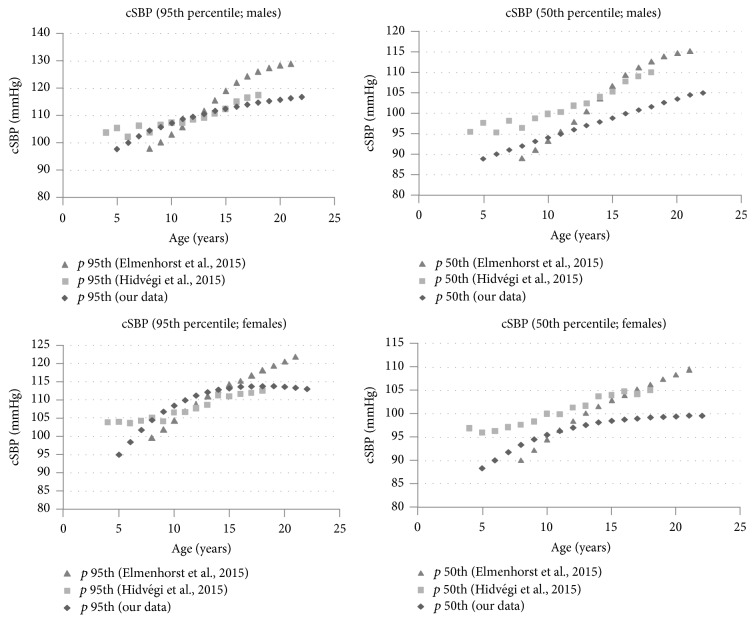
Age specific aortic systolic blood pressure (cSBP) percentiles 95th and 50th obtained in males and females of the Argentinean population compared with European date previously reported.

Our data shows that, in agreement with the two largest data bases of cSBP measurement in children and adolescents with oscillometric techniques, males exhibit higher values of SBP and PP in both central and peripheral arteries [17, 18]. Despite the methodological differences (inclusion and exclusion criteria) in addition to the different devices used between the three studies (see Table 12), it is possible to attempt to compare the cSBP values between our data with the RIs from Hungarian [17] and German pediatric population [18] (Figure 4). In the analyzed population, the 50th percentile corresponding to boys and girls aged between 5 and 22 years shows cSBP values slightly lower than those found by Hidvégi in the Hungarian population using the same technology. Those observations were also valid in boys when 95th percentile was analyzed. However, in girls aged 10 to 17 years, these differences tend to disappear when considering the 95th percentile. On the other hand, in comparison with German database (Mobil-O-Graph System), our data shows that cSBP values in the 50th and the 95th percentiles (both sexes) were slightly higher in children below 10 years, and significantly lower in adolescents over 13 years old than those found by Elmenhorst et al. [18].

**Figure 5:**
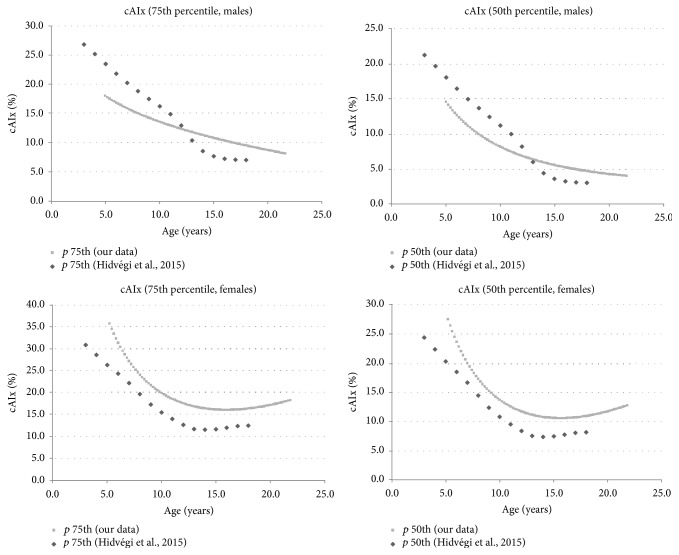
Age specific central (aortic) augmentation index (cAIx) percentiles 75th and 50th obtained in males and females of the Argentinean population compared with Hidvégi et al.'s date previously reported.

On the other hand, values of cAIx show a different behavior with respect to age and gender. As was describe above, our data show that females have higher cAIx values than males (Table 1). These findings are in accordance with the published data of Hidvégi et al. who proved that after 15 years the AIx is higher in females than in males [17]. These authors reported that, in early childhood, the values of AIx are high; however it decreases gradually with age in both genders in ages between 12 and 15 years, increasing afterwards. Moreover, the increased cAIx detected in early childhood cannot be caused by the shorter return time, which is determined by a shorter aortic length [17]. In Figure 5, we compared cAIx percentiles 50th and 75th corresponding ton males and females of the Argentinean population, with those reported by Hidvégi et al. [17]. It is noteworthy that females of both populations exhibit similar cAIx curves, while Argentinean males have lower values than the European cohort before 12 y.o. and higher after ages over 13 y.o.

### 4.1. Methodological Considerations and Limitations

In this observational study a noninvasive occlusive-oscillometric device to assess arterial function parameters in children and adolescents was used. This validated system provides central aortic BP values that have shown significant correlation with values of cSBP invasively assessed [6, 23]. On the other hand, the use of an arm cuff facilitates epidemiological studies performed in large cohort including children and adolescents [14, 17, 33]. Moreover, the Arteriograph device allows simultaneous quantification of Ao-PWV, AIx, cSBP, and peripheral BP, and the procedure takes only 2-3 min and is well tolerated even in preschool children who experienced only a little discomfort, similar to that observed during noninvasive BP measurement [14].

The lack of standardization in methodologies used to obtain RIs and/or normal values for cAIx, cSBP, and other noninvasive obtained arterial parameters makes difficult to compare different populations. In our research, we used the statistical and methodological approach described by the* Reference Values for Arterial Measurements Collaboration Group* [27, 28]. This is not a minor point, since this strategy allows us to compare our data with other databases available around the world.

In this observational study, we used a cross-sectional design; consequently, the relationship between cSBP and cAIx with age should be interpreted with caution. However, generation of these RIs should be generated taking advantage approaches like that used in this research.

## 5. Conclusion

This study, performed in healthy children, adolescents, and young adults from Argentina with ages 5 to 22 y.o., provides the first RIs and percentile curves of cSBP and cAIx. Additionally, specific body height-related cAIx percentiles are reported for the analyzed population. The RIs and percentiles contribute to the knowledge of arterial dynamic evolution along the normal aging process and the interpretation of data obtained in clinical research and daily clinical practice.

## Figures and Tables

**Table 1:** Children and adolescents characteristics.

	All (1038)				Male (*n* = 576)				Female (*n* = 462)				*p* value (male versus female)
	MV	SD	Min	Max	MV	SD	Min	Max	MV	SD	Min	Max	
Age (years)	15.35	3.15	5.00	21.92	15.51	2.84	5.00	21.75	15.15	3.49	5.17	21.92	0.068
Body height (cm)	162.67	13.98	102.00	191.00	167.15	13.72	104.00	191.00	157.09	12.20	102.00	178.00	<0.001
Body weight (Kg)	57.00	14.14	15.00	96.00	61.39	14.70	15.00	96.00	51.51	11.22	18.00	75.00	<0.001
BMI (Kg./m^2^)	21.17	3.11	7.90	29.40	21.60	3.14	13.60	29.40	20.64	2.98	7.90	27.82	<0.001
Jugulo -symphysis distance (cm)	47.48	4.96	28.00	74.00	49.13	4.87	28.00	74.00	45.44	4.27	28.00	56.00	<0.001
Heart rate (b.p.m.)	68.43	12.17	44.00	113.00	65.49	11.83	44.00	104.00	72.09	11.60	44.00	113.00	<0.001
Peripheral SBP (mmHg)	111.81	9.58	81.00	135.00	114.01	9.64	89.00	135.00	109.06	8.78	81.00	134.00	<0.001
Peripheral MBP (mmHg)	79.07	7.49	8.00	102.00	79.24	7.68	8.00	98.00	78.86	7.25	61.00	102.00	0.412
Peripheral DBP (mmHg)	62.71	7.51	3.00	89.00	61.97	7.17	36.00	82.00	63.63	7.84	3.00	89.00	<0.001
Peripheral PP (mmHg)	48.96	8.29	.49	86.00	51.99	8.12	31.00	86.00	45.17	6.81	.49	70.00	<0.001
Central (aortic) SBP (mmHg)	98.86	8.39	75.20	124.90	99.35	8.40	78.00	124.90	98.24	8.36	75.20	121.00	0.035
Central (aortic) PP (mmHg)	36.26	5.88	21.80	62.90	37.43	5.72	21.80	62.90	34.78	5.75	23.20	58.60	<0.001
Peripheral (brachial) AIx (%)	−1.34	7.01	−85.00	.60	−1.64	8.12	−85.00	.29	−.96	5.31	−84.00	.60	0.122
cAIx (%)	9.20	8.46	−12.00	45.90	6.67	7.40	−7.00	36.00	12.35	8.64	−12.00	45.90	<0.001
cAIx HR75 (%)	9.51	8.49	−12.34	45.61	7.12	7.45	−7.82	36.53	12.49	8.76	−12.34	45.61	<0.001
Return time (ms)	173.47	21.17	14.00	310.00	178.91	19.43	85.00	230.00	166.68	21.32	14.00	310.00	<0.001
Ejection duration (ms)	299.06	22.73	185.00	385.00	297.90	21.09	185.00	360.00	300.49	24.59	190.00	385.00	0.068
Ao-PWV (m/s)	5.50	.63	3.40	7.70	5.51	.64	3.40	7.70	5.48	.62	3.90	7.70	0.607

BMI: body mass index. SBP, MBP, DBP, and PP: systolic, mean, diastolic, and pulse blood pressure, respectively. AIx: augmentation index. cAIx and cAIx HR75: central (aortic) augmentation index net value and normalized for heart rate equal 75 beats/minute, respectively. Ao-PWV: Aortic pulse wave velocity. Min and Max: minimal and maximal value, respectively. A *p* < 0.05 was considered statistically significant.

**Table 2:** Association between aortic characteristics and anthropometric and haemodynamic parameters.

		Central (aortic) SBP (mmHg)	cAIx (%)	Age (years)
Sex (0: male; 1: female)	R	−.066	.334	−.057
	P	.035	.000	.068
Age (years)	R	.263	−.188	1.000
	P	.000	.000	.000
Body height (cm)	R	.238	−.371	.678
	P	.000	.000	.000
Body weight (Kg)	R	.318	−.362	.617
	P	.000	.000	.000
BMI (Kg/m^2^)	R	.320	−.215	.414
	P	.000	.000	.000
Yugulo-symphysis distance (cm)	R	.242	−.378	.656
	P	.000	.000	.000
Heart rate (b.p.m.)	R	.054	.005	−.358
	P	.079	.881	.000
Peripheral SBP (mmHg)	R	.892	−.216	.308
	P	.000	.000	.000
Peripheral MBP (mmHg)	R	.863	−.016	.293
	P	.000	.617	.000
Peripheral DBP (mmHg)	R	.720	.107	.236
	P	.000	.001	.000
Peripheral PP (mmHg)	R	.385	−.336	.131
	P	.000	.000	.000
Central (aortic) SBP (mmHg)	R	1.000	.164	.263
	P	.000	.000	.000
Central (aortic) PP (mmHg)	R	.525	.180	.040
	P	.000	.000	.202
Peripheral (brachial) AIx	R	−.018	.079	−.079
	P	.571	.011	.011
cAIx (%)	R	.164	1.000	−.188
	P	.000	.000	.000
cAIx HR75 (%)	R	.162	.997	−.160
	P	.000	.000	.000
Ejection duration (ms)	R	−.026	.207	.096
	P	.396	.000	.002
Return time (ms)	R	−.118	−.327	.152
	P	.000	.000	.000
Ao-PWV (m/s)	R	.374	.010	.456
	P	.000	.757	.000

BMI: body mass index. SBP, MBP, DBP, and PP: systolic, mean, diastolic, and pulse blood pressure, respectively. AIx: augmentation index. cAIx and cAIx HR75: central (aortic) augmentation index net value and normalized for heart rate equal 75 beats/minute, respectively. Ao-PWV: aortic pulse wave velocity. A *p* < 0.05 was considered statistically significant.

**(a):** 

		cSBP (mMHg): before adjustment			cSBP (mmHg): after adjustment by covariates					Covariates appearing in the model are evaluated at the following values					Levene's test	Homogeneity of regression slopes test	Adjusted *R* ^2^
		MV	SD	*p*	MV	SE	95% CI lower bound	95% CI upper bound	*p*	Age (years)	Body height (cm)	Body weight (Kg)	Yugulo-Symphysis distance (cm)	Heart rate (b.p.m.)			
Model 1	Male	99.3517	8.39824	<0.001	99.241	.337	98.579	99.903	0.090	15.35	------	------	------	------	0.773	0.002	0.070
	Female	98.2448	8.35555		98.383	.377	97.643	99.122									
Model 2	Male	99.3517	8.39824	<0.001	98.813	.350	98.125	99.500	0.852	15.35	162.67	57.00	47.48	------	0.797	0.011	0.113
	Female	98.2448	8.35555		98.917	.397	98.139	99.696									
Model 3	Male	99.3517	8.39824	<0.001	99.616	.337	98.954	100.278	0.724	15.35	------	------	------	68.43	0.724	0.001	0.101
	Female	98.2448	8.35555		97.916	.378	97.173	98.658									
Model 4	Male	99.3517	8.39824	<0.001	99.061	.345	98.385	99.737	0.415	15.35	162.67	57.00	47.48	68.43	0.859	0.005	0.151
	Female	98.2448	8.35555		98.607	.391	97.841	99.374									

**(b):** 

		cAIx: before adjustment			cAIx: after adjustment by covariates					Covariates appearing in the model are evaluated at the following values					Levene's test	Homogeneity of regression slopes test	Adjusted *R* ^2^
		MV	SD	*p*	MV	SE	95% CI lower bound	95% CI upper bound	*p*	Age (years)	Body Height (cm)	Body weight (Kg)	Yugulo-Symphysis distance (cm)	Heart rate (b.p.m.)			
Model 1	Male	6.6737	7.40231	<0.001	6.747	.327	6.104	7.389	<0.001	15.35	------	------	------	------	<0.001	0.228	0.138
	Female	12.3474	8.64323		12.257	.366	11.539	12.974									
Model 2	Male	6.6737	7.40231	<0.001	7.72	.337	7.059	8.380	<0.001	15.35	162.67	57.00	47.4846	------	<0.001	0.158	0.193
	Female	12.3474	8.64323		11.043	.381	10.295	11.792									
Model 3	Male	6.6737	7.40231	<0.001	6.411	.328	5.767	7.055	<0.001	15.35	------	------	------	68.43	0.020	0.008	0.163
	Female	12.3474	8.64323		12.675	.368	11.953	13.397									
Model 4	Male	6.6737	7.40231	<0.001	7.447	.329	6.802	8.093	<0.001	15.35	162.67	57.00	47.4846	68.43	0.005	0.006	0.238
	Female	12.3474	8.64323		11.383	.373	10.651	12.115									

cSBP: central (aortic) systolic blood pressure. MV: mean value: SD and SE: standard deviation and error, respectively. CI: confidence interval. cAIx: central (aortic) augmentation index (net value). A *p* < 0.05 was considered statistically significant.

**Table 4:** Age-related reference intervals (RIs) for central (aortic) systolic blood pressure for the entire population (*n* = 1038).

Age (years)	1th	2.5th	5th	10th	25th	50th	75th	90th	95th	97.5th	99th
5.0	77.6	79.2	80.7	82.3	85.1	88.3	91.6	94.6	96.5	98.1	100.0
6.0	77.5	79.4	81.0	83.0	86.3	90.0	93.9	97.5	99.7	101.7	104.0
7.0	77.5	79.6	81.5	83.6	87.3	91.5	95.9	99.9	102.4	104.6	107.2
8.0	77.7	79.9	81.9	84.2	88.2	92.8	97.5	102.0	104.7	107.1	109.9
9.0	77.9	80.3	82.4	84.8	89.0	93.9	99.0	103.7	106.6	109.1	112.2
10.0	78.2	80.7	82.9	85.4	89.8	94.9	100.2	105.1	108.2	110.8	114.0
11.0	78.6	81.2	83.4	86.0	90.5	95.8	101.3	106.4	109.5	112.3	115.6
12.0	79.0	81.6	83.9	86.6	91.2	96.6	102.2	107.4	110.7	113.5	116.9
13.0	79.5	82.1	84.5	87.2	91.9	97.4	103.1	108.4	111.7	114.6	118.0
14.0	80.0	82.7	85.0	87.8	92.5	98.0	103.8	109.2	112.5	115.4	118.9
15.0	80.5	83.2	85.6	88.3	93.1	98.7	104.5	109.9	113.2	116.2	119.7
16.0	81.1	83.8	86.1	88.9	93.7	99.3	105.1	110.5	113.8	116.7	120.3
17.0	81.7	84.4	86.7	89.5	94.3	99.8	105.6	111.0	114.3	117.2	120.7
18.0	82.3	85.0	87.3	90.1	94.8	100.3	106.0	111.4	114.7	117.6	121.1
19.0	83.0	85.6	87.9	90.6	95.3	100.8	106.5	111.8	115.0	117.9	121.3
20.0	83.6	86.2	88.5	91.2	95.9	101.2	106.8	112.1	115.3	118.1	121.5
21.0	84.3	86.9	89.1	91.8	96.4	101.7	107.2	112.3	115.5	118.2	121.6
21.8	84.8	87.4	89.6	92.3	96.8	102.0	107.4	112.5	115.6	118.3	121.6

**Table 5:** Age-related reference intervals (RIs) for central (aortic) systolic blood pressure for males (*n* = 576).

Age (years)	1th	2.5th	5th	10th	25th	50th	75th	90th	95th	97.5th	99th
5.0	76.4	78.4	80.1	82.1	85.3	89.0	92.5	95.8	97.7	99.3	101.2
6.0	75.4	77.7	79.7	82.0	85.8	90.0	94.2	98.0	100.2	102.2	104.4
7.0	74.8	77.4	79.6	82.2	86.4	91.1	95.7	99.9	102.4	104.5	107.0
8.0	74.7	77.5	79.8	82.6	87.1	92.1	97.1	101.5	104.2	106.5	109.1
9.0	74.8	77.8	80.3	83.1	87.9	93.1	98.4	103.0	105.8	108.2	111.0
10.0	75.2	78.2	80.8	83.8	88.7	94.1	99.5	104.3	107.2	109.7	112.6
11.0	75.8	78.9	81.5	84.5	89.6	95.1	100.6	105.5	108.5	111.0	113.9
12.0	76.5	79.6	82.3	85.4	90.5	96.1	101.7	106.6	109.6	112.2	115.1
13.0	77.4	80.5	83.2	86.3	91.4	97.0	102.7	107.6	110.6	113.2	116.2
14.0	78.4	81.5	84.2	87.3	92.4	98.0	103.6	108.6	111.6	114.2	117.1
15.0	79.4	82.5	85.2	88.3	93.3	98.9	104.5	109.5	112.4	115.0	118.0
16.0	80.6	83.6	86.3	89.3	94.3	99.9	105.4	110.3	113.2	115.8	118.7
17.0	81.8	84.8	87.4	90.4	95.3	100.8	106.3	111.1	114.0	116.5	119.4
18.0	83.1	86.0	88.6	91.5	96.4	101.7	107.1	111.9	114.7	117.1	120.0
19.0	84.4	87.3	89.8	92.7	97.4	102.7	107.9	112.6	115.3	117.7	120.5
20.0	85.8	88.6	91.0	93.8	98.5	103.6	108.7	113.2	116.0	118.3	121.0
21.0	87.2	90.0	92.3	95.0	99.5	104.5	109.5	113.9	116.5	118.8	121.5
21.6	88.1	90.8	93.1	95.8	100.2	105.1	109.9	114.3	116.9	119.1	121.7

**Table 6:** Age-related reference intervals (RIs) for central (aortic) systolic blood pressure for females (*n* = 462).

Age (years)	1th	2.5th	5th	10th	25th	50th	75th	90th	95th	97.5th	99th
5.2	79.5	80.8	82.0	83.4	85.7	88.3	91.0	93.5	95.0	96.3	97.9
6.0	79.2	80.9	82.3	84.0	86.8	90.0	93.4	96.4	98.3	100.0	101.9
7.0	79.1	81.0	82.6	84.6	88.0	91.8	95.8	99.5	101.7	103.7	106.0
8.0	79.0	81.1	83.0	85.2	88.9	93.3	97.8	101.9	104.5	106.7	109.4
9.0	79.0	81.3	83.3	85.7	89.8	94.5	99.4	103.9	106.7	109.2	112.1
10.0	79.1	81.5	83.7	86.2	90.5	95.5	100.7	105.5	108.5	111.1	114.3
11.0	79.2	81.7	84.0	86.6	91.1	96.3	101.8	106.8	110.0	112.7	116.0
12.0	79.4	82.0	84.3	87.0	91.6	97.0	102.6	107.9	111.1	114.0	117.4
13.0	79.6	82.3	84.6	87.4	92.1	97.6	103.4	108.7	112.0	115.0	118.5
14.0	79.9	82.5	84.9	87.7	92.5	98.1	103.9	109.4	112.7	115.7	119.2
15.0	80.1	82.8	85.2	88.0	92.9	98.5	104.4	109.8	113.2	116.2	119.8
16.0	80.4	83.1	85.5	88.3	93.2	98.8	104.7	110.2	113.6	116.6	120.1
17.0	80.7	83.4	85.8	88.6	93.5	99.1	104.9	110.4	113.7	116.7	120.3
18.0	81.1	83.8	86.1	88.9	93.7	99.2	105.0	110.4	113.8	116.7	120.3
19.0	81.4	84.1	86.4	89.2	93.9	99.4	105.1	110.4	113.7	116.6	120.1
20.0	81.8	84.4	86.7	89.4	94.1	99.5	105.1	110.3	113.6	116.4	119.8
21.0	82.2	84.8	87.0	89.7	94.2	99.5	105.0	110.1	113.3	116.1	119.4
21.8	82.5	85.1	87.3	89.9	94.3	99.5	104.9	109.9	113.0	115.7	119.0

**Table 7:** Age-related reference intervals (RIs) for central (aortic) augmentation index (cAIx) for the entire population (*n* = 1038).

Age (years)	1th	2.5th	5th	10th	25th	50th	75th	90th	95th	97.5th	99th
5.0	1.8	4.5	7.1	10.2	16.0	23.3	31.4	39.4	44.5	49.1	54.7
6.0	−0.1	2.4	4.7	7.5	12.8	19.3	26.7	34.0	38.6	42.8	48.0
7.0	−1.5	0.8	2.9	5.5	10.3	16.4	23.3	30.0	34.3	38.3	43.0
8.0	−2.6	−0.5	1.5	3.9	8.5	14.2	20.6	27.0	31.1	34.8	39.4
9.0	−3.4	−1.5	0.4	2.7	7.0	12.5	18.7	24.8	28.7	32.3	36.6
10.0	−4.1	−2.3	−0.5	1.7	5.9	11.2	17.1	23.0	26.8	30.3	34.5
11.0	−4.7	−2.9	−1.2	1.0	5.0	10.1	15.9	21.7	25.4	28.8	32.9
12.0	−5.2	−3.4	−1.8	0.3	4.3	9.3	15.0	20.7	24.3	27.7	31.7
13.0	−5.6	−3.8	−2.2	−0.2	3.7	8.7	14.3	19.9	23.6	26.9	30.9
14.0	−5.9	−4.2	−2.6	−0.6	3.3	8.2	13.8	19.4	23.0	26.3	30.4
15.0	−6.1	−4.5	−2.9	−0.9	2.9	7.8	13.4	19.1	22.7	26.0	30.1
16.0	−6.4	−4.7	−3.2	−1.1	2.7	7.6	13.2	18.9	22.5	25.9	30.0
17.0	−6.6	−4.9	−3.4	−1.3	2.5	7.4	13.1	18.8	22.5	25.9	30.0
18.0	−6.7	−5.1	−3.5	−1.5	2.4	7.4	13.1	18.9	22.6	26.1	30.3
19.0	−6.8	−5.2	−3.6	−1.6	2.3	7.4	13.2	19.1	22.9	26.4	30.6
20.0	−6.9	−5.3	−3.7	−1.6	2.3	7.4	13.4	19.3	23.2	26.8	31.1
21.0	−7.0	−5.4	−3.8	−1.7	2.3	7.6	13.6	19.7	23.7	27.3	31.8
21.8	−7.1	−5.4	−3.8	−1.7	2.4	7.7	13.8	20.1	24.1	27.8	32.3

**Table 8:** Age-related reference intervals (RIs) for central (aortic) augmentation index (cAIx) for males (*n* = 576).

Age (years)	1th	2.5th	5th	10th	25th	50th	75th	90th	95th	97.5th	99th
5.0	4.6	6.0	7.2	8.7	11.4	14.6	18.1	21.4	23.4	25.3	27.5
6.0	1.2	2.7	4.1	5.8	8.9	12.7	16.9	21.0	23.6	26.0	28.8
7.0	−1.1	0.4	1.9	3.7	7.0	11.2	15.9	20.6	23.6	26.3	29.6
8.0	−2.6	−1.1	0.3	2.1	5.6	10.0	15.1	20.2	23.4	26.4	30.0
9.0	−3.6	−2.2	−0.8	1.0	4.5	9.0	14.3	19.7	23.1	26.3	30.2
10.0	−4.3	−3.0	−1.6	0.1	3.6	8.2	13.6	19.1	22.7	26.0	30.0
11.0	−4.8	−3.6	−2.3	−0.5	2.9	7.5	13.0	18.6	22.2	25.6	29.7
12.0	−5.2	−4.0	−2.7	−1.0	2.4	7.0	12.4	18.0	21.7	25.1	29.2
13.0	−5.4	−4.3	−3.1	−1.4	1.9	6.5	11.9	17.5	21.1	24.5	28.7
14.0	−5.6	−4.5	−3.3	−1.7	1.6	6.0	11.4	16.9	20.5	23.8	27.9
15.0	−5.6	−4.6	−3.5	−1.9	1.3	5.6	10.9	16.3	19.8	23.1	27.2
16.0	−5.7	−4.7	−3.5	−2.0	1.1	5.3	10.4	15.7	19.2	22.4	26.3
17.0	−5.7	−4.7	−3.6	−2.1	0.9	5.0	10.0	15.1	18.5	21.6	25.4
18.0	−5.6	−4.6	−3.6	−2.1	0.8	4.8	9.6	14.5	17.7	20.7	24.4
19.0	−5.6	−4.6	−3.5	−2.1	0.7	4.6	9.2	13.9	17.0	19.9	23.4
20.0	−5.4	−4.5	−3.4	−2.1	0.7	4.4	8.8	13.3	16.3	19.0	22.3
21.0	−5.3	−4.3	−3.3	−2.0	0.7	4.2	8.4	12.7	15.5	18.1	21.3
21.6	−5.2	−4.2	−3.2	−1.9	0.7	4.1	8.2	12.4	15.1	17.6	20.6

**Table 9:** Age-related reference intervals (RIs) for central (aortic) augmentation index (cAIx) for females (*n* = 462).

Age (years)	1th	2.5th	5th	10th	25th	50th	75th	90th	95th	97.5th	99th
5.2	3.5	6.8	9.7	13.3	19.8	27.5	35.8	43.7	48.6	53.0	58.2
6.0	1.4	4.4	7.2	10.5	16.5	23.7	31.4	38.8	43.4	47.5	52.4
7.0	−0.5	2.2	4.8	7.8	13.4	20.1	27.3	34.1	38.4	42.3	46.8
8.0	−1.9	0.6	3.0	5.9	11.1	17.4	24.1	30.6	34.7	38.3	42.6
9.0	−2.9	−0.5	1.7	4.4	9.3	15.3	21.8	28.0	31.8	35.3	39.4
10.0	−3.7	−1.4	0.7	3.4	8.1	13.8	20.0	25.9	29.6	33.0	36.9
11.0	−4.2	−2.0	0.1	2.6	7.1	12.7	18.6	24.4	28.0	31.2	35.0
12.0	−4.5	−2.4	−0.4	2.0	6.4	11.8	17.7	23.2	26.7	29.9	33.6
13.0	−4.7	−2.7	−0.7	1.7	6.0	11.3	17.0	22.4	25.9	28.9	32.6
14.0	−4.8	−2.8	−0.9	1.5	5.7	10.9	16.5	21.9	25.2	28.3	31.9
15.0	−4.8	−2.8	−0.9	1.4	5.6	10.7	16.3	21.6	24.9	27.9	31.4
16.0	−4.8	−2.7	−0.8	1.5	5.6	10.7	16.2	21.4	24.7	27.7	31.2
17.0	−4.6	−2.6	−0.7	1.6	5.8	10.8	16.3	21.5	24.8	27.7	31.2
18.0	−4.4	−2.3	−0.5	1.9	6.0	11.0	16.5	21.7	25.0	27.9	31.4
19.0	−4.1	−2.0	−0.1	2.2	6.3	11.4	16.8	22.1	25.3	28.2	31.7
20.0	−3.7	−1.7	0.3	2.6	6.8	11.8	17.3	22.5	25.8	28.7	32.2
21.0	−3.3	−1.2	0.7	3.1	7.3	12.4	17.9	23.1	26.4	29.3	32.8
21.8	−3.0	−0.8	1.1	3.5	7.7	12.9	18.4	23.7	27.0	29.9	33.4

**Table 10:** Body height-related reference intervals (RIs) for central (aortic) augmentation index (cAIx) for males (*n* = 576).

Body height (cm)	1th	2.5th	5th	10th	25th	50th	75th	90th	95th	97.5th	99th
104	5.06	6.36	7.54	8.95	11.43	14.41	17.59	20.62	22.51	24.20	26.23
105	4.79	6.12	7.32	8.76	11.31	14.35	17.62	20.74	22.69	24.44	26.52
110	3.54	4.97	6.26	7.83	10.64	14.03	17.70	21.23	23.45	25.44	27.83
115	2.38	3.87	5.24	6.91	9.93	13.61	17.63	21.52	23.98	26.19	28.85
120	1.31	2.85	4.26	6.01	9.18	13.10	17.41	21.61	24.28	26.69	29.59
125	0.33	1.88	3.33	5.12	8.41	12.52	17.07	21.54	24.39	26.96	30.08
130	−0.58	0.97	2.43	4.25	7.62	11.87	16.62	21.31	24.31	27.03	30.32
135	−1.41	0.11	1.57	3.40	6.82	11.17	16.07	20.93	24.06	26.89	30.33
140	−2.18	−0.69	0.75	2.57	6.01	10.42	15.43	20.43	23.65	26.58	30.14
145	−2.88	−1.44	−0.03	1.76	5.19	9.63	14.71	19.80	23.09	26.09	29.75
150	−3.51	−2.13	−0.77	0.99	4.37	8.81	13.91	19.07	22.41	25.46	29.18
155	−4.09	−2.78	−1.48	0.23	3.56	7.96	13.06	18.24	21.61	24.69	28.46
160	−4.61	−3.38	−2.14	−0.49	2.76	7.09	12.16	17.33	20.70	23.79	27.58
165	−5.07	−3.93	−2.76	−1.18	1.96	6.21	11.21	16.34	19.70	22.79	26.57
170	−5.49	−4.44	−3.34	−1.84	1.19	5.32	10.23	15.30	18.62	21.68	25.44
175	−5.85	−4.90	−3.88	−2.47	0.43	4.43	9.23	14.20	17.47	20.50	24.21
180	−6.16	−5.31	−4.38	−3.06	−0.31	3.54	8.20	13.06	16.27	19.24	22.89
185	−6.42	−5.68	−4.83	−3.61	−1.02	2.66	7.16	11.88	15.02	17.91	21.50
190	−6.63	−6.01	−5.25	−4.13	−1.71	1.80	6.12	10.68	13.72	16.54	20.03
191	−6.67	−6.07	−5.33	−4.23	−1.84	1.62	5.91	10.44	13.46	16.26	19.73

**Table 11:** Body height-related reference intervals (RIs) for central (aortic) augmentation index (cAIx) for females (*n* = 462).

Body height (cm)	1th	2.5th	5th	10th	25th	50th	75th	90th	95th	97.5th	99th
102	−1.63	1.21	3.82	7.03	12.82	19.86	27.47	34.76	39.32	43.40	48.27
105	−1.07	1.71	4.27	7.40	13.02	19.83	27.18	34.20	38.60	42.52	47.20
110	−0.36	2.34	4.80	7.80	13.17	19.65	26.61	33.25	37.40	41.09	45.50
115	0.10	2.71	5.09	7.98	13.12	19.31	25.95	32.26	36.20	39.71	43.89
120	0.32	2.85	5.15	7.93	12.88	18.83	25.19	31.24	35.01	38.37	42.36
125	0.31	2.76	4.99	7.69	12.48	18.22	24.36	30.19	33.82	37.06	40.91
130	0.09	2.48	4.64	7.26	11.92	17.50	23.46	29.12	32.64	35.78	39.52
135	−0.32	2.01	4.12	6.68	11.22	16.67	22.49	28.02	31.47	34.53	38.19
140	−0.90	1.37	3.44	5.95	10.40	15.75	21.47	26.91	30.30	33.32	36.91
145	−1.63	0.60	2.62	5.09	9.47	14.75	20.40	25.78	29.14	32.13	35.69
150	−2.48	−0.30	1.69	4.11	8.45	13.67	19.29	24.64	27.99	30.97	34.52
155	−3.44	−1.31	0.65	3.04	7.34	12.54	18.14	23.49	26.84	29.83	33.40
160	−4.48	−2.40	−0.47	1.89	6.16	11.35	16.96	22.34	25.71	28.72	32.31
165	−5.57	−3.55	−1.66	0.68	4.92	10.11	15.75	21.18	24.58	27.63	31.27
170	−6.70	−4.74	−2.89	−0.58	3.63	8.83	14.52	20.01	23.46	26.56	30.26
175	−7.83	−5.95	−4.15	−1.88	2.31	7.53	13.28	18.84	22.36	25.51	29.29
178	−8.49	−6.67	−4.90	−2.67	1.50	6.74	12.52	18.14	21.70	24.89	28.72

**Table 12:** Subjects characteristics described for Europeans (Germany and Hungary) and our studied populations.

	Elmenhorst et al. 2015		Hidvégi et al. 2015		Díaz et al. (our work)	
*(1) Characteristics of the sample*						
Number of subjects	1445 (females: 715; males: 730)		4619 (females: 2130; males: 2489)		1038 (females: 462; males: 576)	
Subjects precedence	Living in Germany (Munich)		Living in Hungary		Living in Argentina (Tandil)	
Age interval	8–22 years		3–18 years		5–21 years	
Recruitment strategy	Prospective (school-based setting)		Prospective (nursery, elementary, and secondary schools)		Prospective (nursery, elementary, and secondary schools)	
Communities	Urban/rural		-------		Urban/rural	
Inclusion criteria	Absence of known CV; even if treated		Apparently healthy; Caucasian; without medication		Asymptomatic. (1) None had history of CV, pulmonary, or renal disease; (2) normal brachial BP at the time of examination; (3) none were taking medications, (4) all had normal levels of glycaemia, cholesterol, and triglycerides	
Exclusion criteria	Hypertensive BP (<18 years: according to guideline; ≥18 years: 140/90 mmHg); obesity		BMI: 3th–97th percentile; SBP and DBP: 5th–95th percentile		Current and past smokers, diabetic, obese subjects, hypertensive subjects or subjects with averaged high brachial BP levels at the time of the study were excluded	

*(2) Aortic BP levels and waveform measurement*						
Device	Mobil-O-Graph, I.E.M., Stolberg, Germany		Arteriograph, TensioMed Ltd., Budapest, Hungary		Arteriograph, TensioMed Ltd., Budapest, Hungary	
Measurement conditions	Supine position after at least 5–10 min of rest		Supine position after 2-3 minutes of rest		Supine position after at least 10 min of rest	

* (3) Characteristics of the subjects*	MV	SD	MV	SD	MV	SD

Female						
Age (years)	13.8	2.9	10.5	4.8	15.1	3.5
Body height (cm)	157.0	11.6	142.7	22.4	157.1	12.2
Body weight (Kg)	48.8	12.5	37.8	15.6	51.5	11.2
BMI (Kg/m^2^)	19.5	3.2	17.4	2.5	20.6	3.0
pSBP (mmHg)	113.5	7.5	110.9	6.0	109.1	8.8
pDBP (mmHg)	66.0	6.8	64.7	3.0	63.6	7.8
pMBP (mmHg)	81.8	6.2	80.2	3.9	78.9	7.3
cSBP (mmHg)	100.5	8.0	100.3	3.3	98.2	8.4
Ao-PWV (m/s)	4.6	0.3	---	---	5.5	0.6
Heart rate (b.p.m.)	---	---	82.6	7.5	72.1	11.6
Yugulo-Symphysis distance (cm)	---	---	42.0	6.6	45.4	4.3
cAIx (%)	---	---	11.9	4.4	12.3	8.6
Male						
Age (years)	13.1	2.6	10.5	4.8	15.5	2.8
Body height (cm)	158.7	15.6	146.3	25.7	167.2	13.7
Body weight (Kg)	49.1	15.2	40.5	18.5	61.4	14.7
BMI (Kg/m^2^)	19.0	3.1	17.6	2.4	21.6	3.1
pSBP (mmHg)	114.5	8.3	113.3	8.4	114.0	9.6
pDBP (mmHg)	65.1	6.8	64.7	2.6	62.0	7.2
pMBP (mmHg)	81.6	6.3	81.1	4.3	79.2	7.7
cSBP (mmHg)	100.8	9.7	101.2	4.8	99.4	8.4
Ao-PWV (m/s)	4.7	0.4			5.5	0.6
Heart rate (b.p.m.)	---	---	79.1	8.7	65.5	11.8
Yugulo-Symphysis distance (cm)	---	---	43.9	7.6	49.1	4.9
cAIx (%)	---	---	10.2	4.5	6.7	7.4
All						
Age (years)	13.4	2.8	---	---	15.3	3.1
Body height (cm)	----	----	---	---	162.7	14.0
Body weight (Kg)	----	----	---	---	57.0	14.1
pSBP (mmHg)	114.0	7.9	---	---	111.8	9.6
cSBP (mmHg)	100.7	8.9	---	---	98.9	8.4
Ao-PWV (m/s)	4.7	0.3	---	---	5.5	0.6

BMI: body mass index. pSBP, pMBP, and pDBP: peripheral (brachial) systolic, mean, and diastolic blood pressure, respectively. cAIx: central (aortic) augmentation index. Ao-PWV: aortic pulse wave velocity. CV: cardiovascular. MV: mean value. SD: standard deviation. cSBP: central (aortic) systolic blood pressure, respectively. Data from [18] were directly obtained from average population values published in their manuscript. Data from [17] were obtained from averaging the disaggregated values per year of life (age), for each sex.

## Supplementary Materials

Supplementary Materials Table A: age-related reference intervals (RIs) for central (aortic) systolic blood pressure for the entire population (*n* = 1038). Table B: age-related reference intervals (RIs) for central (aortic) systolic blood pressure for males (*n* = 576). Table C: age-related reference intervals (RIs) for central (aortic) systolic blood pressure for females (*n* = 462). Table D: age-related reference intervals (RIs) for central (aortic) augmentation index (cAIx) for the entire population (*n* = 1038). Table E: age-related reference intervals (RIs) for central (aortic) augmentation index (cAIx) for males (*n* = 576). Table F: age-related reference intervals (RIs) for central (aortic) augmentation index (cAIx) for females (*n* = 462). Table G: body height-related reference intervals (RIs) for central (aortic) augmentation index (cAIx) for males (*n* = 576). Table H: body height-related reference intervals (RIs) for central (aortic) augmentation index (cAIx) for females (*n* = 462).
