# Supplementary material for: Reference Intervals of Central Aortic Blood Pressure and Augmentation Index Assessed with an Oscillometric Device in Healthy Children, Adolescents, and Young Adults from Argentina
Source: Int J Hypertens. 2018 Apr 23;2018:1469651. doi: 10.1155/2018/1469651 (PMC5937421; doi:10.1155/2018/1469651)
Supplement: Supplementary Materials — Table A: age-related reference intervals (RIs) for central (aortic) systolic blood pressure for the entire population (n = 1038). Table B: age-related reference intervals (RIs) for central (aortic) systolic blood pressure for males (n = 576). Table C: age-related reference intervals (RIs) for central (aortic) systolic blood pressure for females (n = 462). Table D: age-related reference intervals (RIs) for central (aortic) augmentation index (cAIx) for the entire population (n = 1038). Table E: age-related reference intervals (RIs) for central (aortic) augmentation index (cAIx) for males (n = 576). Table F: age-related reference intervals (RIs) for central (aortic) augmentation index (cAIx) for females (n = 462). Table G: body height-related reference intervals (RIs) for central (aortic) augmentation index (cAIx) for males (n = 576). Table H: body height-related reference intervals (RIs) for central (aortic) augmentation index (cAIx) for females (n = 462). [file 1469651.f1.pdf]

Table A. Age-related reference intervals (RIs) for central (aortic) systolic blood pressure for the entire population (n=1038)

Age (years)	1 th	2.5 th	5 th	10 th	25 th	50 th	75 th	90 th	95 th	97.5 th	99 th
5.0	77.6	79.2	80.7	82.3	85.1	88.3	91.6	94.6	96.5	98.1	100.0
5.2	77.6	79.3	80.7	82.4	85.3	88.7	92.1	95.2	97.2	98.9	100.9
5.4	77.6	79.3	80.8	82.6	85.6	89.0	92.6	95.9	97.9	99.6	101.7
5.6	77.5	79.3	80.9	82.7	85.8	89.4	93.0	96.4	98.5	100.3	102.5
5.8	77.5	79.4	81.0	82.8	86.0	89.7	93.5	97.0	99.1	101.0	103.3
6.0	77.5	79.4	81.0	83.0	86.3	90.0	93.9	97.5	99.7	101.7	104.0
6.2	77.5	79.4	81.1	83.1	86.5	90.3	94.4	98.1	100.3	102.3	104.7
6.4	77.5	79.5	81.2	83.2	86.7	90.6	94.8	98.6	100.9	102.9	105.4
6.6	77.5	79.5	81.3	83.3	86.9	90.9	95.1	99.0	101.4	103.5	106.0
6.8	77.5	79.6	81.4	83.5	87.1	91.2	95.5	99.5	101.9	104.1	106.6
7.0	77.5	79.6	81.5	83.6	87.3	91.5	95.9	99.9	102.4	104.6	107.2
7.2	77.6	79.7	81.5	83.7	87.5	91.8	96.2	100.4	102.9	105.2	107.8
7.4	77.6	79.7	81.6	83.8	87.6	92.0	96.6	100.8	103.4	105.7	108.4
7.6	77.6	79.8	81.7	84.0	87.8	92.3	96.9	101.2	103.8	106.2	108.9
7.8	77.6	79.9	81.8	84.1	88.0	92.5	97.2	101.6	104.3	106.6	109.4
8.0	77.7	79.9	81.9	84.2	88.2	92.8	97.5	102.0	104.7	107.1	109.9
8.2	77.7	80.0	82.0	84.3	88.4	93.0	97.8	102.3	105.1	107.5	110.4
8.4	77.8	80.1	82.1	84.5	88.5	93.2	98.1	102.7	105.5	107.9	110.9
8.6	77.8	80.1	82.2	84.6	88.7	93.5	98.4	103.0	105.9	108.4	111.3
8.8	77.9	80.2	82.3	84.7	88.9	93.7	98.7	103.3	106.2	108.7	111.8
9.0	77.9	80.3	82.4	84.8	89.0	93.9	99.0	103.7	106.6	109.1	112.2
9.2	78.0	80.4	82.5	84.9	89.2	94.1	99.2	104.0	106.9	109.5	112.6
9.4	78.0	80.5	82.6	85.1	89.4	94.3	99.5	104.3	107.2	109.9	113.0
9.6	78.1	80.5	82.7	85.2	89.5	94.5	99.7	104.6	107.6	110.2	113.3
9.8	78.2	80.6	82.8	85.3	89.7	94.7	100.0	104.8	107.9	110.5	113.7
10.0	78.2	80.7	82.9	85.4	89.8	94.9	100.2	105.1	108.2	110.8	114.0
10.2	78.3	80.8	83.0	85.5	90.0	95.1	100.4	105.4	108.4	111.2	114.4
10.4	78.4	80.9	83.1	85.7	90.1	95.3	100.6	105.6	108.7	111.5	114.7
10.6	78.5	81.0	83.2	85.8	90.3	95.4	100.9	105.9	109.0	111.7	115.0
10.8	78.5	81.1	83.3	85.9	90.4	95.6	101.1	106.1	109.3	112.0	115.3
11.0	78.6	81.2	83.4	86.0	90.5	95.8	101.3	106.4	109.5	112.3	115.6
11.2	78.7	81.2	83.5	86.1	90.7	96.0	101.5	106.6	109.8	112.6	115.9
11.4	78.8	81.3	83.6	86.3	90.8	96.1	101.7	106.8	110.0	112.8	116.2
11.6	78.9	81.4	83.7	86.4	91.0	96.3	101.8	107.0	110.2	113.1	116.4
11.8	78.9	81.5	83.8	86.5	91.1	96.5	102.0	107.2	110.5	113.3	116.7
12.0	79.0	81.6	83.9	86.6	91.2	96.6	102.2	107.4	110.7	113.5	116.9
12.2	79.1	81.7	84.0	86.7	91.4	96.8	102.4	107.6	110.9	113.7	117.2
12.4	79.2	81.8	84.1	86.8	91.5	96.9	102.6	107.8	111.1	114.0	117.4
12.6	79.3	81.9	84.2	87.0	91.6	97.1	102.7	108.0	111.3	114.2	117.6
12.8	79.4	82.0	84.3	87.1	91.8	97.2	102.9	108.2	111.5	114.4	117.8
13.0	79.5	82.1	84.5	87.2	91.9	97.4	103.1	108.4	111.7	114.6	118.0
13.2	79.6	82.2	84.6	87.3	92.0	97.5	103.2	108.5	111.8	114.7	118.2
13.4	79.7	82.3	84.7	87.4	92.1	97.6	103.4	108.7	112.0	114.9	118.4
13.6	79.8	82.5	84.8	87.5	92.3	97.8	103.5	108.9	112.2	115.1	118.6
13.8	79.9	82.6	84.9	87.7	92.4	97.9	103.7	109.0	112.3	115.3	118.8
14.0	80.0	82.7	85.0	87.8	92.5	98.0	103.8	109.2	112.5	115.4	118.9
14.2	80.1	82.8	85.1	87.9	92.6	98.2	103.9	109.3	112.6	115.6	119.1
14.4	80.2	82.9	85.2	88.0	92.8	98.3	104.1	109.5	112.8	115.7	119.2
14.6	80.3	83.0	85.3	88.1	92.9	98.4	104.2	109.6	112.9	115.9	119.4
14.8	80.4	83.1	85.5	88.2	93.0	98.5	104.3	109.7	113.1	116.0	119.5
15.0	80.5	83.2	85.6	88.3	93.1	98.7	104.5	109.9	113.2	116.2	119.7
15.2	80.7	83.3	85.7	88.5	93.2	98.8	104.6	110.0	113.3	116.3	119.8
15.4	80.8	83.4	85.8	88.6	93.4	98.9	104.7	110.1	113.4	116.4	119.9
15.6	80.9	83.6	85.9	88.7	93.5	99.0	104.8	110.2	113.6	116.5	120.0
15.8	81.0	83.7	86.0	88.8	93.6	99.1	104.9	110.3	113.7	116.6	120.1
16.0	81.1	83.8	86.1	88.9	93.7	99.3	105.1	110.5	113.8	116.7	120.3
16.2	81.2	83.9	86.3	89.0	93.8	99.4	105.2	110.6	113.9	116.9	120.4
16.4	81.3	84.0	86.4	89.2	93.9	99.5	105.3	110.7	114.0	117.0	120.5
16.6	81.5	84.1	86.5	89.3	94.0	99.6	105.4	110.8	114.1	117.1	120.6
16.8	81.6	84.3	86.6	89.4	94.2	99.7	105.5	110.9	114.2	117.1	120.6
17.0	81.7	84.4	86.7	89.5	94.3	99.8	105.6	111.0	114.3	117.2	120.7
17.2	81.8	84.5	86.8	89.6	94.4	99.9	105.7	111.1	114.4	117.3	120.8
17.4	81.9	84.6	87.0	89.7	94.5	100.0	105.8	111.1	114.5	117.4	120.9
17.6	82.1	84.7	87.1	89.8	94.6	100.1	105.9	111.2	114.5	117.5	120.9
17.8	82.2	84.9	87.2	90.0	94.7	100.2	106.0	111.3	114.6	117.5	121.0
18.0	82.3	85.0	87.3	90.1	94.8	100.3	106.0	111.4	114.7	117.6	121.1
18.2	82.4	85.1	87.4	90.2	94.9	100.4	106.1	111.5	114.8	117.7	121.1
18.4	82.6	85.2	87.6	90.3	95.0	100.5	106.2	111.5	114.8	117.7	121.2
18.6	82.7	85.3	87.7	90.4	95.1	100.6	106.3	111.6	114.9	117.8	121.2
18.8	82.8	85.5	87.8	90.5	95.2	100.7	106.4	111.7	115.0	117.9	121.3
19.0	83.0	85.6	87.9	90.6	95.3	100.8	106.5	111.8	115.0	117.9	121.3
19.2	83.1	85.7	88.0	90.8	95.5	100.9	106.5	111.8	115.1	118.0	121.4
19.4	83.2	85.8	88.2	90.9	95.6	101.0	106.6	111.9	115.1	118.0	121.4
19.6	83.3	86.0	88.3	91.0	95.7	101.1	106.7	111.9	115.2	118.0	121.4
19.8	83.5	86.1	88.4	91.1	95.8	101.2	106.8	112.0	115.2	118.1	121.5
20.0	83.6	86.2	88.5	91.2	95.9	101.2	106.8	112.1	115.3	118.1	121.5
20.2	83.7	86.4	88.6	91.3	96.0	101.3	106.9	112.1	115.3	118.1	121.5
20.4	83.9	86.5	88.8	91.5	96.1	101.4	107.0	112.2	115.4	118.2	121.5
20.6	84.0	86.6	88.9	91.6	96.2	101.5	107.0	112.2	115.4	118.2	121.5
20.8	84.2	86.7	89.0	91.7	96.3	101.6	107.1	112.3	115.4	118.2	121.5
21.0	84.3	86.9	89.1	91.8	96.4	101.7	107.2	112.3	115.5	118.2	121.6
21.2	84.4	87.0	89.3	91.9	96.5	101.7	107.2	112.3	115.5	118.3	121.6
21.4	84.6	87.1	89.4	92.0	96.6	101.8	107.3	112.4	115.5	118.3	121.6
21.6	84.7	87.3	89.5	92.1	96.7	101.9	107.3	112.4	115.5	118.3	121.6
21.8	84.8	87.4	89.6	92.3	96.8	102.0	107.4	112.5	115.6	118.3	121.6

Table B. Age-related reference intervals (RIs) for central (aortic) systolic blood pressure for males (n=576)

Age (years)	1 th	2.5 th	5 th	10 th	25 th	50 th	75 th	90 th	95 th	97.5 th	99 th
5.0	76.4	78.4	80.1	82.1	85.3	89.0	92.5	95.8	97.7	99.3	101.2
5.2	76.2	78.2	80.0	82.0	85.4	89.2	92.9	96.2	98.2	99.9	101.9
5.4	75.9	78.1	79.9	82.0	85.5	89.4	93.3	96.7	98.8	100.5	102.6
5.6	75.7	77.9	79.8	82.0	85.6	89.6	93.6	97.1	99.3	101.1	103.2
5.8	75.5	77.8	79.8	82.0	85.7	89.8	93.9	97.6	99.8	101.6	103.8
6.0	75.4	77.7	79.7	82.0	85.8	90.0	94.2	98.0	100.2	102.2	104.4
6.2	75.2	77.6	79.7	82.0	85.9	90.3	94.6	98.4	100.7	102.7	105.0
6.4	75.1	77.5	79.6	82.0	86.0	90.5	94.9	98.8	101.1	103.2	105.5
6.6	75.0	77.5	79.6	82.1	86.2	90.7	95.2	99.2	101.6	103.6	106.0
6.8	74.9	77.4	79.6	82.1	86.3	90.9	95.5	99.5	102.0	104.1	106.5
7.0	74.8	77.4	79.6	82.2	86.4	91.1	95.7	99.9	102.4	104.5	107.0
7.2	74.8	77.4	79.7	82.3	86.5	91.3	96.0	100.2	102.8	104.9	107.5
7.4	74.7	77.4	79.7	82.3	86.7	91.5	96.3	100.6	103.1	105.3	107.9
7.6	74.7	77.4	79.7	82.4	86.8	91.7	96.6	100.9	103.5	105.7	108.3
7.8	74.7	77.4	79.8	82.5	87.0	91.9	96.8	101.2	103.9	106.1	108.7
8.0	74.7	77.5	79.8	82.6	87.1	92.1	97.1	101.5	104.2	106.5	109.1
8.2	74.7	77.5	79.9	82.7	87.3	92.3	97.4	101.9	104.5	106.8	109.5
8.4	74.7	77.6	80.0	82.8	87.4	92.5	97.6	102.2	104.9	107.2	109.9
8.6	74.7	77.6	80.1	82.9	87.6	92.7	97.9	102.4	105.2	107.5	110.3
8.8	74.8	77.7	80.2	83.0	87.7	92.9	98.1	102.7	105.5	107.9	110.6
9.0	74.8	77.8	80.3	83.1	87.9	93.1	98.4	103.0	105.8	108.2	111.0
9.2	74.9	77.8	80.4	83.2	88.0	93.3	98.6	103.3	106.1	108.5	111.3
9.4	75.0	77.9	80.5	83.4	88.2	93.5	98.8	103.6	106.4	108.8	111.6
9.6	75.0	78.0	80.6	83.5	88.4	93.7	99.1	103.8	106.7	109.1	111.9
9.8	75.1	78.1	80.7	83.6	88.5	93.9	99.3	104.1	106.9	109.4	112.3
10.0	75.2	78.2	80.8	83.8	88.7	94.1	99.5	104.3	107.2	109.7	112.6
10.2	75.3	78.4	81.0	83.9	88.9	94.3	99.7	104.6	107.5	110.0	112.8
10.4	75.4	78.5	81.1	84.1	89.0	94.5	100.0	104.8	107.7	110.2	113.1
10.6	75.5	78.6	81.2	84.2	89.2	94.7	100.2	105.1	108.0	110.5	113.4
10.8	75.7	78.7	81.4	84.4	89.4	94.9	100.4	105.3	108.2	110.7	113.7
11.0	75.8	78.9	81.5	84.5	89.6	95.1	100.6	105.5	108.5	111.0	113.9
11.2	75.9	79.0	81.7	84.7	89.7	95.3	100.8	105.8	108.7	111.2	114.2
11.4	76.1	79.2	81.8	84.9	89.9	95.5	101.0	106.0	108.9	111.5	114.4
11.6	76.2	79.3	82.0	85.0	90.1	95.7	101.3	106.2	109.2	111.7	114.7
11.8	76.4	79.5	82.2	85.2	90.3	95.9	101.5	106.4	109.4	111.9	114.9
12.0	76.5	79.6	82.3	85.4	90.5	96.1	101.7	106.6	109.6	112.2	115.1
12.2	76.7	79.8	82.5	85.6	90.6	96.3	101.9	106.8	109.8	112.4	115.4
12.4	76.9	80.0	82.7	85.7	90.8	96.5	102.1	107.0	110.0	112.6	115.6
12.6	77.0	80.2	82.8	85.9	91.0	96.7	102.3	107.3	110.2	112.8	115.8
12.8	77.2	80.3	83.0	86.1	91.2	96.9	102.5	107.5	110.4	113.0	116.0
13.0	77.4	80.5	83.2	86.3	91.4	97.0	102.7	107.6	110.6	113.2	116.2
13.2	77.6	80.7	83.4	86.5	91.6	97.2	102.8	107.8	110.8	113.4	116.4
13.4	77.8	80.9	83.6	86.7	91.8	97.4	103.0	108.0	111.0	113.6	116.6
13.6	78.0	81.1	83.8	86.9	92.0	97.6	103.2	108.2	111.2	113.8	116.8
13.8	78.2	81.3	84.0	87.1	92.2	97.8	103.4	108.4	111.4	114.0	117.0
14.0	78.4	81.5	84.2	87.3	92.4	98.0	103.6	108.6	111.6	114.2	117.1
14.2	78.6	81.7	84.4	87.4	92.5	98.2	103.8	108.8	111.8	114.3	117.3
14.4	78.8	81.9	84.6	87.6	92.7	98.4	104.0	109.0	111.9	114.5	117.5
14.6	79.0	82.1	84.8	87.9	92.9	98.6	104.2	109.1	112.1	114.7	117.7
14.8	79.2	82.3	85.0	88.1	93.1	98.8	104.3	109.3	112.3	114.8	117.8
15.0	79.4	82.5	85.2	88.3	93.3	98.9	104.5	109.5	112.4	115.0	118.0
15.2	79.6	82.8	85.4	88.5	93.5	99.1	104.7	109.7	112.6	115.2	118.1
15.4	79.9	83.0	85.6	88.7	93.7	99.3	104.9	109.8	112.8	115.3	118.3
15.6	80.1	83.2	85.8	88.9	93.9	99.5	105.0	110.0	112.9	115.5	118.4
15.8	80.3	83.4	86.1	89.1	94.1	99.7	105.2	110.2	113.1	115.6	118.6
16.0	80.6	83.6	86.3	89.3	94.3	99.9	105.4	110.3	113.2	115.8	118.7
16.2	80.8	83.9	86.5	89.5	94.5	100.1	105.6	110.5	113.4	115.9	118.9
16.4	81.0	84.1	86.7	89.7	94.7	100.3	105.7	110.6	113.6	116.1	119.0
16.6	81.3	84.3	87.0	90.0	94.9	100.4	105.9	110.8	113.7	116.2	119.1
16.8	81.5	84.6	87.2	90.2	95.1	100.6	106.1	110.9	113.9	116.4	119.3
17.0	81.8	84.8	87.4	90.4	95.3	100.8	106.3	111.1	114.0	116.5	119.4
17.2	82.0	85.1	87.6	90.6	95.5	101.0	106.4	111.3	114.1	116.6	119.5
17.4	82.3	85.3	87.9	90.8	95.8	101.2	106.6	111.4	114.3	116.8	119.6
17.6	82.5	85.5	88.1	91.1	96.0	101.4	106.8	111.6	114.4	116.9	119.8
17.8	82.8	85.8	88.4	91.3	96.2	101.6	106.9	111.7	114.6	117.0	119.9
18.0	83.1	86.0	88.6	91.5	96.4	101.7	107.1	111.9	114.7	117.1	120.0
18.2	83.3	86.3	88.8	91.7	96.6	101.9	107.3	112.0	114.8	117.3	120.1
18.4	83.6	86.5	89.1	92.0	96.8	102.1	107.4	112.1	115.0	117.4	120.2
18.6	83.9	86.8	89.3	92.2	97.0	102.3	107.6	112.3	115.1	117.5	120.3
18.8	84.1	87.0	89.6	92.4	97.2	102.5	107.7	112.4	115.2	117.6	120.4
19.0	84.4	87.3	89.8	92.7	97.4	102.7	107.9	112.6	115.3	117.7	120.5
19.2	84.7	87.6	90.0	92.9	97.6	102.9	108.1	112.7	115.5	117.9	120.6
19.4	84.9	87.8	90.3	93.1	97.8	103.0	108.2	112.8	115.6	118.0	120.7
19.6	85.2	88.1	90.5	93.4	98.0	103.2	108.4	113.0	115.7	118.1	120.8
19.8	85.5	88.3	90.8	93.6	98.3	103.4	108.5	113.1	115.8	118.2	120.9
20.0	85.8	88.6	91.0	93.8	98.5	103.6	108.7	113.2	116.0	118.3	121.0
20.2	86.1	88.9	91.3	94.1	98.7	103.8	108.8	113.4	116.1	118.4	121.1
20.4	86.3	89.1	91.5	94.3	98.9	104.0	109.0	113.5	116.2	118.5	121.2
20.6	86.6	89.4	91.8	94.5	99.1	104.1	109.2	113.6	116.3	118.6	121.3
20.8	86.9	89.7	92.1	94.8	99.3	104.3	109.3	113.8	116.4	118.7	121.4
21.0	87.2	90.0	92.3	95.0	99.5	104.5	109.5	113.9	116.5	118.8	121.5
21.2	87.5	90.2	92.6	95.3	99.7	104.7	109.6	114.0	116.6	118.9	121.5
21.4	87.8	90.5	92.8	95.5	100.0	104.9	109.8	114.1	116.7	119.0	121.6
21.6	88.1	90.8	93.1	95.8	100.2	105.1	109.9	114.3	116.9	119.1	121.7

Table C. Age-related reference intervals (RIs) for central (aortic) systolic blood pressure for females (n=462)

Age (years)	1 th	2.5 th	5 th	10 th	25 th	50 th	75 th	90 th	95 th	97.5 th	99 th
5.2	79.5	80.8	82.0	83.4	85.7	88.3	91.0	93.5	95.0	96.3	97.9
5.4	79.4	80.8	82.1	83.5	86.0	88.8	91.7	94.3	95.9	97.3	99.0
5.6	79.3	80.8	82.1	83.7	86.3	89.2	92.3	95.0	96.7	98.2	100.0
5.8	79.3	80.9	82.2	83.8	86.5	89.6	92.8	95.8	97.5	99.1	101.0
6.0	79.2	80.9	82.3	84.0	86.8	90.0	93.4	96.4	98.3	100.0	101.9
6.2	79.2	80.9	82.4	84.1	87.0	90.4	93.9	97.1	99.1	100.8	102.8
6.4	79.1	80.9	82.4	84.2	87.3	90.8	94.4	97.7	99.8	101.6	103.7
6.6	79.1	80.9	82.5	84.4	87.5	91.2	94.9	98.3	100.4	102.3	104.5
6.8	79.1	80.9	82.6	84.5	87.7	91.5	95.4	98.9	101.1	103.0	105.3
7.0	79.1	81.0	82.6	84.6	88.0	91.8	95.8	99.5	101.7	103.7	106.0
7.2	79.0	81.0	82.7	84.7	88.2	92.1	96.2	100.0	102.3	104.4	106.8
7.4	79.0	81.0	82.8	84.8	88.4	92.4	96.6	100.5	102.9	105.0	107.5
7.6	79.0	81.1	82.9	85.0	88.6	92.7	97.0	101.0	103.4	105.6	108.1
7.8	79.0	81.1	82.9	85.1	88.8	93.0	97.4	101.5	104.0	106.2	108.8
8.0	79.0	81.1	83.0	85.2	88.9	93.3	97.8	101.9	104.5	106.7	109.4
8.2	79.0	81.2	83.1	85.3	89.1	93.5	98.1	102.3	105.0	107.3	110.0
8.4	79.0	81.2	83.1	85.4	89.3	93.8	98.4	102.8	105.4	107.8	110.5
8.6	79.0	81.2	83.2	85.5	89.5	94.0	98.8	103.2	105.9	108.3	111.1
8.8	79.0	81.3	83.3	85.6	89.6	94.3	99.1	103.5	106.3	108.7	111.6
9.0	79.0	81.3	83.3	85.7	89.8	94.5	99.4	103.9	106.7	109.2	112.1
9.2	79.0	81.3	83.4	85.8	89.9	94.7	99.6	104.3	107.1	109.6	112.6
9.4	79.0	81.4	83.5	85.9	90.1	94.9	99.9	104.6	107.5	110.0	113.0
9.6	79.0	81.4	83.5	86.0	90.2	95.1	100.2	104.9	107.8	110.4	113.5
9.8	79.1	81.5	83.6	86.1	90.3	95.3	100.4	105.2	108.2	110.8	113.9
10.0	79.1	81.5	83.7	86.2	90.5	95.5	100.7	105.5	108.5	111.1	114.3
10.2	79.1	81.6	83.7	86.3	90.6	95.7	100.9	105.8	108.8	111.5	114.7
10.4	79.1	81.6	83.8	86.3	90.7	95.8	101.1	106.1	109.1	111.8	115.0
10.6	79.2	81.7	83.8	86.4	90.9	96.0	101.4	106.3	109.4	112.1	115.4
10.8	79.2	81.7	83.9	86.5	91.0	96.2	101.6	106.6	109.7	112.4	115.7
11.0	79.2	81.7	84.0	86.6	91.1	96.3	101.8	106.8	110.0	112.7	116.0
11.2	79.2	81.8	84.0	86.7	91.2	96.5	102.0	107.1	110.2	113.0	116.3
11.4	79.3	81.8	84.1	86.8	91.3	96.6	102.1	107.3	110.5	113.3	116.6
11.6	79.3	81.9	84.2	86.8	91.4	96.8	102.3	107.5	110.7	113.5	116.9
11.8	79.4	81.9	84.2	86.9	91.5	96.9	102.5	107.7	110.9	113.8	117.1
12.0	79.4	82.0	84.3	87.0	91.6	97.0	102.6	107.9	111.1	114.0	117.4
12.2	79.4	82.0	84.4	87.1	91.7	97.1	102.8	108.1	111.3	114.2	117.6
12.4	79.5	82.1	84.4	87.1	91.8	97.3	102.9	108.2	111.5	114.4	117.9
12.6	79.5	82.2	84.5	87.2	91.9	97.4	103.1	108.4	111.7	114.6	118.1
12.8	79.6	82.2	84.5	87.3	92.0	97.5	103.2	108.6	111.9	114.8	118.3
13.0	79.6	82.3	84.6	87.4	92.1	97.6	103.4	108.7	112.0	115.0	118.5
13.2	79.7	82.3	84.7	87.4	92.2	97.7	103.5	108.9	112.2	115.1	118.6
13.4	79.7	82.4	84.7	87.5	92.3	97.8	103.6	109.0	112.3	115.3	118.8
13.6	79.8	82.4	84.8	87.6	92.4	97.9	103.7	109.1	112.5	115.4	119.0
13.8	79.8	82.5	84.8	87.6	92.4	98.0	103.8	109.2	112.6	115.6	119.1
14.0	79.9	82.5	84.9	87.7	92.5	98.1	103.9	109.4	112.7	115.7	119.2
14.2	79.9	82.6	85.0	87.8	92.6	98.2	104.0	109.5	112.8	115.8	119.4
14.4	80.0	82.7	85.0	87.8	92.7	98.3	104.1	109.6	112.9	115.9	119.5
14.6	80.0	82.7	85.1	87.9	92.7	98.3	104.2	109.7	113.0	116.0	119.6
14.8	80.1	82.8	85.2	88.0	92.8	98.4	104.3	109.8	113.1	116.1	119.7
15.0	80.1	82.8	85.2	88.0	92.9	98.5	104.4	109.8	113.2	116.2	119.8
15.2	80.2	82.9	85.3	88.1	92.9	98.6	104.4	109.9	113.3	116.3	119.9
15.4	80.2	83.0	85.3	88.2	93.0	98.6	104.5	110.0	113.4	116.4	119.9
15.6	80.3	83.0	85.4	88.2	93.1	98.7	104.6	110.1	113.4	116.4	120.0
15.8	80.4	83.1	85.5	88.3	93.1	98.7	104.6	110.1	113.5	116.5	120.1
16.0	80.4	83.1	85.5	88.3	93.2	98.8	104.7	110.2	113.6	116.6	120.1
16.2	80.5	83.2	85.6	88.4	93.2	98.9	104.7	110.2	113.6	116.6	120.2
16.4	80.6	83.3	85.6	88.5	93.3	98.9	104.8	110.3	113.6	116.6	120.2
16.6	80.6	83.3	85.7	88.5	93.3	99.0	104.8	110.3	113.7	116.7	120.2
16.8	80.7	83.4	85.8	88.6	93.4	99.0	104.9	110.3	113.7	116.7	120.3
17.0	80.7	83.4	85.8	88.6	93.5	99.1	104.9	110.4	113.7	116.7	120.3
17.2	80.8	83.5	85.9	88.7	93.5	99.1	104.9	110.4	113.8	116.7	120.3
17.4	80.9	83.6	85.9	88.7	93.6	99.1	105.0	110.4	113.8	116.7	120.3
17.6	80.9	83.6	86.0	88.8	93.6	99.2	105.0	110.4	113.8	116.8	120.3
17.8	81.0	83.7	86.1	88.9	93.6	99.2	105.0	110.4	113.8	116.8	120.3
18.0	81.1	83.8	86.1	88.9	93.7	99.2	105.0	110.4	113.8	116.7	120.3
18.2	81.2	83.8	86.2	89.0	93.7	99.3	105.1	110.5	113.8	116.7	120.2
18.4	81.2	83.9	86.2	89.0	93.8	99.3	105.1	110.5	113.8	116.7	120.2
18.6	81.3	84.0	86.3	89.1	93.8	99.3	105.1	110.5	113.8	116.7	120.2
18.8	81.4	84.0	86.4	89.1	93.9	99.4	105.1	110.4	113.7	116.7	120.1
19.0	81.4	84.1	86.4	89.2	93.9	99.4	105.1	110.4	113.7	116.6	120.1
19.2	81.5	84.2	86.5	89.2	93.9	99.4	105.1	110.4	113.7	116.6	120.0
19.4	81.6	84.2	86.5	89.3	94.0	99.4	105.1	110.4	113.7	116.6	120.0
19.6	81.7	84.3	86.6	89.3	94.0	99.4	105.1	110.4	113.6	116.5	119.9
19.8	81.7	84.4	86.7	89.4	94.0	99.5	105.1	110.4	113.6	116.5	119.9
20.0	81.8	84.4	86.7	89.4	94.1	99.5	105.1	110.3	113.6	116.4	119.8
20.2	81.9	84.5	86.8	89.5	94.1	99.5	105.1	110.3	113.5	116.3	119.7
20.4	82.0	84.6	86.8	89.5	94.1	99.5	105.1	110.3	113.5	116.3	119.6
20.6	82.1	84.6	86.9	89.6	94.2	99.5	105.0	110.2	113.4	116.2	119.6
20.8	82.1	84.7	87.0	89.6	94.2	99.5	105.0	110.2	113.3	116.1	119.5
21.0	82.2	84.8	87.0	89.7	94.2	99.5	105.0	110.1	113.3	116.1	119.4
21.2	82.3	84.8	87.1	89.7	94.3	99.5	105.0	110.1	113.2	116.0	119.3
21.4	82.4	84.9	87.1	89.8	94.3	99.5	105.0	110.0	113.2	115.9	119.2
21.6	82.4	85.0	87.2	89.8	94.3	99.5	104.9	110.0	113.1	115.8	119.1
21.8	82.5	85.1	87.3	89.9	94.3	99.5	104.9	109.9	113.0	115.7	119.0

Table D. Age-related reference intervals (RIs) for central (aortic) augmentation index (cAIx) for the entire population (n=1038)											
Age (years)	1 th	2.5 th	5 th	10 th	25 th	50 th	75 th	90 th	95 th	97.5 th	99 th
5.0	1.8	4.5	7.1	10.2	16.0	23.3	31.4	39.4	44.5	49.1	54.7
5.2	1.4	4.1	6.5	9.6	15.3	22.4	30.3	38.2	43.2	47.7	53.2
5.4	1.0	3.6	6.0	9.0	14.6	21.6	29.3	37.0	41.9	46.3	51.7
5.6	0.6	3.2	5.5	8.5	14.0	20.8	28.4	35.9	40.7	45.1	50.4
5.8	0.3	2.8	5.1	8.0	13.3	20.0	27.5	34.9	39.7	43.9	49.1
6.0	-0.1	2.4	4.7	7.5	12.8	19.3	26.7	34.0	38.6	42.8	48.0
6.2	-0.4	2.0	4.3	7.1	12.2	18.7	25.9	33.1	37.7	41.8	46.9
6.4	-0.7	1.7	3.9	6.6	11.7	18.1	25.2	32.2	36.8	40.8	45.8
6.6	-1.0	1.4	3.5	6.2	11.2	17.5	24.5	31.5	35.9	39.9	44.8
6.8	-1.2	1.1	3.2	5.9	10.8	16.9	23.9	30.7	35.1	39.1	43.9
7.0	-1.5	0.8	2.9	5.5	10.3	16.4	23.3	30.0	34.3	38.3	43.0
7.2	-1.7	0.5	2.6	5.1	9.9	15.9	22.7	29.3	33.6	37.5	42.2
7.4	-1.9	0.2	2.3	4.8	9.5	15.5	22.1	28.7	32.9	36.8	41.4
7.6	-2.2	0.0	2.0	4.5	9.2	15.0	21.6	28.1	32.3	36.1	40.7
7.8	-2.4	-0.3	1.7	4.2	8.8	14.6	21.1	27.6	31.7	35.5	40.0
8.0	-2.6	-0.5	1.5	3.9	8.5	14.2	20.6	27.0	31.1	34.8	39.4
8.2	-2.8	-0.7	1.2	3.7	8.2	13.8	20.2	26.5	30.6	34.3	38.7
8.4	-2.9	-0.9	1.0	3.4	7.9	13.5	19.8	26.0	30.1	33.7	38.2
8.6	-3.1	-1.1	0.8	3.2	7.6	13.1	19.4	25.6	29.6	33.2	37.6
8.8	-3.3	-1.3	0.6	2.9	7.3	12.8	19.0	25.2	29.1	32.7	37.1
9.0	-3.4	-1.5	0.4	2.7	7.0	12.5	18.7	24.8	28.7	32.3	36.6
9.2	-3.6	-1.6	0.2	2.5	6.8	12.2	18.3	24.4	28.3	31.8	36.1
9.4	-3.7	-1.8	0.0	2.3	6.5	11.9	18.0	24.0	27.9	31.4	35.7
9.6	-3.9	-2.0	-0.2	2.1	6.3	11.7	17.7	23.7	27.5	31.0	35.3
9.8	-4.0	-2.1	-0.3	1.9	6.1	11.4	17.4	23.3	27.2	30.6	34.9
10.0	-4.1	-2.3	-0.5	1.7	5.9	11.2	17.1	23.0	26.8	30.3	34.5
10.2	-4.2	-2.4	-0.6	1.6	5.7	10.9	16.8	22.7	26.5	30.0	34.1
10.4	-4.4	-2.5	-0.8	1.4	5.5	10.7	16.6	22.4	26.2	29.6	33.8
10.6	-4.5	-2.7	-0.9	1.3	5.3	10.5	16.4	22.2	25.9	29.3	33.5
10.8	-4.6	-2.8	-1.1	1.1	5.2	10.3	16.1	21.9	25.7	29.1	33.2
11.0	-4.7	-2.9	-1.2	1.0	5.0	10.1	15.9	21.7	25.4	28.8	32.9
11.2	-4.8	-3.0	-1.3	0.8	4.8	9.9	15.7	21.5	25.2	28.5	32.7
11.4	-4.9	-3.1	-1.4	0.7	4.7	9.8	15.5	21.2	24.9	28.3	32.4
11.6	-5.0	-3.2	-1.5	0.6	4.5	9.6	15.3	21.0	24.7	28.1	32.2
11.8	-5.1	-3.3	-1.7	0.5	4.4	9.4	15.2	20.9	24.5	27.9	32.0
12.0	-5.2	-3.4	-1.8	0.3	4.3	9.3	15.0	20.7	24.3	27.7	31.7
12.2	-5.2	-3.5	-1.9	0.2	4.1	9.2	14.8	20.5	24.2	27.5	31.6
12.4	-5.3	-3.6	-2.0	0.1	4.0	9.0	14.7	20.3	24.0	27.3	31.4
12.6	-5.4	-3.7	-2.0	0.0	3.9	8.9	14.5	20.2	23.8	27.2	31.2
12.8	-5.5	-3.8	-2.1	-0.1	3.8	8.8	14.4	20.1	23.7	27.0	31.1
13.0	-5.6	-3.8	-2.2	-0.2	3.7	8.7	14.3	19.9	23.6	26.9	30.9
13.2	-5.6	-3.9	-2.3	-0.3	3.6	8.6	14.2	19.8	23.4	26.8	30.8
13.4	-5.7	-4.0	-2.4	-0.3	3.5	8.5	14.1	19.7	23.3	26.6	30.7
13.6	-5.8	-4.1	-2.5	-0.4	3.4	8.4	14.0	19.6	23.2	26.5	30.6
13.8	-5.8	-4.1	-2.5	-0.5	3.3	8.3	13.9	19.5	23.1	26.4	30.5
14.0	-5.9	-4.2	-2.6	-0.6	3.3	8.2	13.8	19.4	23.0	26.3	30.4
14.2	-5.9	-4.3	-2.7	-0.6	3.2	8.1	13.7	19.3	22.9	26.3	30.3
14.4	-6.0	-4.3	-2.7	-0.7	3.1	8.0	13.6	19.2	22.9	26.2	30.2
14.6	-6.0	-4.4	-2.8	-0.8	3.0	8.0	13.6	19.2	22.8	26.1	30.2
14.8	-6.1	-4.4	-2.9	-0.8	3.0	7.9	13.5	19.1	22.7	26.1	30.1
15.0	-6.1	-4.5	-2.9	-0.9	2.9	7.8	13.4	19.1	22.7	26.0	30.1
15.2	-6.2	-4.5	-3.0	-0.9	2.9	7.8	13.4	19.0	22.6	26.0	30.0
15.4	-6.2	-4.6	-3.0	-1.0	2.8	7.7	13.3	19.0	22.6	25.9	30.0
15.6	-6.3	-4.6	-3.1	-1.1	2.8	7.7	13.3	18.9	22.6	25.9	30.0
15.8	-6.3	-4.7	-3.1	-1.1	2.7	7.6	13.2	18.9	22.5	25.9	30.0
16.0	-6.4	-4.7	-3.2	-1.1	2.7	7.6	13.2	18.9	22.5	25.9	30.0
16.2	-6.4	-4.8	-3.2	-1.2	2.6	7.5	13.2	18.8	22.5	25.9	30.0
16.4	-6.5	-4.8	-3.2	-1.2	2.6	7.5	13.2	18.8	22.5	25.9	30.0
16.6	-6.5	-4.9	-3.3	-1.3	2.5	7.5	13.1	18.8	22.5	25.9	30.0
16.8	-6.5	-4.9	-3.3	-1.3	2.5	7.5	13.1	18.8	22.5	25.9	30.0
17.0	-6.6	-4.9	-3.4	-1.3	2.5	7.4	13.1	18.8	22.5	25.9	30.0
17.2	-6.6	-5.0	-3.4	-1.4	2.5	7.4	13.1	18.8	22.5	25.9	30.1
17.4	-6.6	-5.0	-3.4	-1.4	2.4	7.4	13.1	18.8	22.5	25.9	30.1
17.6	-6.7	-5.0	-3.5	-1.4	2.4	7.4	13.1	18.8	22.6	26.0	30.2
17.8	-6.7	-5.1	-3.5	-1.5	2.4	7.4	13.1	18.9	22.6	26.0	30.2
18.0	-6.7	-5.1	-3.5	-1.5	2.4	7.4	13.1	18.9	22.6	26.1	30.3
18.2	-6.7	-5.1	-3.5	-1.5	2.3	7.4	13.1	18.9	22.7	26.1	30.3
18.4	-6.8	-5.1	-3.6	-1.5	2.3	7.4	13.1	18.9	22.7	26.2	30.4
18.6	-6.8	-5.2	-3.6	-1.6	2.3	7.4	13.1	19.0	22.8	26.2	30.5
18.8	-6.8	-5.2	-3.6	-1.6	2.3	7.4	13.2	19.0	22.8	26.3	30.6
19.0	-6.8	-5.2	-3.6	-1.6	2.3	7.4	13.2	19.1	22.9	26.4	30.6
19.2	-6.9	-5.2	-3.6	-1.6	2.3	7.4	13.2	19.1	22.9	26.4	30.7
19.4	-6.9	-5.3	-3.7	-1.6	2.3	7.4	13.2	19.2	23.0	26.5	30.8
19.6	-6.9	-5.3	-3.7	-1.6	2.3	7.4	13.3	19.2	23.1	26.6	30.9
19.8	-6.9	-5.3	-3.7	-1.6	2.3	7.4	13.3	19.3	23.2	26.7	31.0
20.0	-6.9	-5.3	-3.7	-1.6	2.3	7.4	13.4	19.3	23.2	26.8	31.1
20.2	-7.0	-5.3	-3.7	-1.7	2.3	7.4	13.4	19.4	23.3	26.9	31.3
20.4	-7.0	-5.3	-3.7	-1.7	2.3	7.5	13.4	19.5	23.4	27.0	31.4
20.6	-7.0	-5.3	-3.7	-1.7	2.3	7.5	13.5	19.5	23.5	27.1	31.5
20.8	-7.0	-5.4	-3.7	-1.7	2.3	7.5	13.5	19.6	23.6	27.2	31.6
21.0	-7.0	-5.4	-3.8	-1.7	2.3	7.6	13.6	19.7	23.7	27.3	31.8
21.2	-7.0	-5.4	-3.8	-1.7	2.3	7.6	13.7	19.8	23.8	27.4	31.9
21.4	-7.1	-5.4	-3.8	-1.7	2.4	7.6	13.7	19.9	23.9	27.6	32.0
21.6	-7.1	-5.4	-3.8	-1.7	2.4	7.7	13.8	20.0	24.0	27.7	32.2
21.8	-7.1	-5.4	-3.8	-1.7	2.4	7.7	13.8	20.1	24.1	27.8	32.3

Table E. Age-related reference intervals (RIs) for central (aortic) augmentation index (cAIx) for males (n=576)

Age (years)	1 th	2.5 th	5 th	10 th	25 th	50 th	75 th	90 th	95 th	97.5 th	99 th
5.0	4.6	6.0	7.2	8.7	11.4	14.6	18.1	21.4	23.4	25.3	27.5
5.2	3.8	5.2	6.5	8.0	10.8	14.2	17.8	21.3	23.5	25.5	27.8
5.4	3.0	4.5	5.8	7.4	10.3	13.8	17.6	21.2	23.5	25.6	28.1
5.6	2.4	3.8	5.2	6.8	9.8	13.4	17.3	21.2	23.6	25.8	28.4
5.8	1.7	3.2	4.6	6.3	9.3	13.0	17.1	21.1	23.6	25.9	28.6
6.0	1.2	2.7	4.1	5.8	8.9	12.7	16.9	21.0	23.6	26.0	28.8
6.2	0.6	2.2	3.6	5.3	8.5	12.4	16.7	20.9	23.6	26.1	29.0
6.4	0.2	1.7	3.1	4.8	8.1	12.1	16.5	20.9	23.6	26.2	29.2
6.6	-0.3	1.2	2.7	4.4	7.7	11.8	16.3	20.8	23.6	26.2	29.4
6.8	-0.7	0.8	2.3	4.0	7.3	11.5	16.1	20.7	23.6	26.3	29.5
7.0	-1.1	0.4	1.9	3.7	7.0	11.2	15.9	20.6	23.6	26.3	29.6
7.2	-1.4	0.1	1.5	3.3	6.7	11.0	15.8	20.5	23.6	26.4	29.7
7.4	-1.7	-0.2	1.2	3.0	6.4	10.7	15.6	20.4	23.6	26.4	29.8
7.6	-2.0	-0.5	0.9	2.7	6.1	10.5	15.4	20.3	23.5	26.4	29.9
7.8	-2.3	-0.8	0.6	2.4	5.8	10.2	15.3	20.3	23.5	26.4	30.0
8.0	-2.6	-1.1	0.3	2.1	5.6	10.0	15.1	20.2	23.4	26.4	30.0
8.2	-2.8	-1.3	0.1	1.9	5.3	9.8	14.9	20.1	23.4	26.4	30.1
8.4	-3.0	-1.6	-0.2	1.6	5.1	9.6	14.8	20.0	23.3	26.4	30.1
8.6	-3.2	-1.8	-0.4	1.4	4.9	9.4	14.6	19.9	23.3	26.4	30.1
8.8	-3.4	-2.0	-0.6	1.2	4.7	9.2	14.5	19.8	23.2	26.3	30.2
9.0	-3.6	-2.2	-0.8	1.0	4.5	9.0	14.3	19.7	23.1	26.3	30.2
9.2	-3.8	-2.4	-1.0	0.8	4.3	8.9	14.2	19.6	23.1	26.2	30.1
9.4	-3.9	-2.5	-1.2	0.6	4.1	8.7	14.0	19.5	23.0	26.2	30.1
9.6	-4.1	-2.7	-1.3	0.4	3.9	8.5	13.9	19.3	22.9	26.1	30.1
9.8	-4.2	-2.8	-1.5	0.3	3.8	8.4	13.8	19.2	22.8	26.1	30.1
10.0	-4.3	-3.0	-1.6	0.1	3.6	8.2	13.6	19.1	22.7	26.0	30.0
10.2	-4.4	-3.1	-1.8	0.0	3.4	8.1	13.5	19.0	22.6	25.9	30.0
10.4	-4.5	-3.2	-1.9	-0.2	3.3	7.9	13.4	18.9	22.5	25.9	29.9
10.6	-4.6	-3.4	-2.0	-0.3	3.2	7.8	13.2	18.8	22.4	25.8	29.9
10.8	-4.7	-3.5	-2.2	-0.4	3.0	7.7	13.1	18.7	22.3	25.7	29.8
11.0	-4.8	-3.6	-2.3	-0.5	2.9	7.5	13.0	18.6	22.2	25.6	29.7
11.2	-4.9	-3.7	-2.4	-0.6	2.8	7.4	12.9	18.5	22.1	25.5	29.6
11.4	-5.0	-3.7	-2.5	-0.8	2.7	7.3	12.8	18.4	22.0	25.4	29.5
11.6	-5.0	-3.8	-2.6	-0.9	2.6	7.2	12.6	18.3	21.9	25.3	29.5
11.8	-5.1	-3.9	-2.6	-0.9	2.5	7.1	12.5	18.1	21.8	25.2	29.4
12.0	-5.2	-4.0	-2.7	-1.0	2.4	7.0	12.4	18.0	21.7	25.1	29.2
12.2	-5.2	-4.0	-2.8	-1.1	2.3	6.8	12.3	17.9	21.6	25.0	29.1
12.4	-5.3	-4.1	-2.9	-1.2	2.2	6.7	12.2	17.8	21.5	24.9	29.0
12.6	-5.3	-4.2	-2.9	-1.3	2.1	6.6	12.1	17.7	21.4	24.7	28.9
12.8	-5.4	-4.2	-3.0	-1.3	2.0	6.5	12.0	17.6	21.2	24.6	28.8
13.0	-5.4	-4.3	-3.1	-1.4	1.9	6.5	11.9	17.5	21.1	24.5	28.7
13.2	-5.4	-4.3	-3.1	-1.5	1.8	6.4	11.8	17.3	21.0	24.4	28.5
13.4	-5.5	-4.3	-3.2	-1.5	1.8	6.3	11.7	17.2	20.9	24.2	28.4
13.6	-5.5	-4.4	-3.2	-1.6	1.7	6.2	11.6	17.1	20.7	24.1	28.2
13.8	-5.5	-4.4	-3.3	-1.6	1.6	6.1	11.5	17.0	20.6	24.0	28.1
14.0	-5.6	-4.5	-3.3	-1.7	1.6	6.0	11.4	16.9	20.5	23.8	27.9
14.2	-5.6	-4.5	-3.3	-1.7	1.5	5.9	11.3	16.7	20.4	23.7	27.8
14.4	-5.6	-4.5	-3.4	-1.8	1.4	5.9	11.2	16.6	20.2	23.6	27.6
14.6	-5.6	-4.5	-3.4	-1.8	1.4	5.8	11.1	16.5	20.1	23.4	27.5
14.8	-5.6	-4.6	-3.4	-1.9	1.3	5.7	11.0	16.4	20.0	23.3	27.3
15.0	-5.6	-4.6	-3.5	-1.9	1.3	5.6	10.9	16.3	19.8	23.1	27.2
15.2	-5.7	-4.6	-3.5	-1.9	1.2	5.6	10.8	16.2	19.7	23.0	27.0
15.4	-5.7	-4.6	-3.5	-2.0	1.2	5.5	10.7	16.0	19.6	22.8	26.8
15.6	-5.7	-4.6	-3.5	-2.0	1.1	5.4	10.6	15.9	19.4	22.7	26.6
15.8	-5.7	-4.6	-3.5	-2.0	1.1	5.4	10.5	15.8	19.3	22.5	26.5
16.0	-5.7	-4.7	-3.5	-2.0	1.1	5.3	10.4	15.7	19.2	22.4	26.3
16.2	-5.7	-4.7	-3.6	-2.1	1.0	5.3	10.3	15.6	19.0	22.2	26.1
16.4	-5.7	-4.7	-3.6	-2.1	1.0	5.2	10.2	15.4	18.9	22.0	25.9
16.6	-5.7	-4.7	-3.6	-2.1	1.0	5.1	10.2	15.3	18.7	21.9	25.7
16.8	-5.7	-4.7	-3.6	-2.1	0.9	5.1	10.1	15.2	18.6	21.7	25.6
17.0	-5.7	-4.7	-3.6	-2.1	0.9	5.0	10.0	15.1	18.5	21.6	25.4
17.2	-5.7	-4.7	-3.6	-2.1	0.9	5.0	9.9	15.0	18.3	21.4	25.2
17.4	-5.7	-4.7	-3.6	-2.1	0.8	4.9	9.8	14.9	18.2	21.2	25.0
17.6	-5.7	-4.7	-3.6	-2.1	0.8	4.9	9.7	14.7	18.0	21.1	24.8
17.8	-5.6	-4.6	-3.6	-2.1	0.8	4.8	9.6	14.6	17.9	20.9	24.6
18.0	-5.6	-4.6	-3.6	-2.1	0.8	4.8	9.6	14.5	17.7	20.7	24.4
18.2	-5.6	-4.6	-3.6	-2.1	0.8	4.7	9.5	14.4	17.6	20.6	24.2
18.4	-5.6	-4.6	-3.6	-2.1	0.7	4.7	9.4	14.3	17.4	20.4	24.0
18.6	-5.6	-4.6	-3.6	-2.1	0.7	4.7	9.3	14.1	17.3	20.2	23.8
18.8	-5.6	-4.6	-3.5	-2.1	0.7	4.6	9.2	14.0	17.2	20.0	23.6
19.0	-5.6	-4.6	-3.5	-2.1	0.7	4.6	9.2	13.9	17.0	19.9	23.4
19.2	-5.5	-4.6	-3.5	-2.1	0.7	4.5	9.1	13.8	16.9	19.7	23.2
19.4	-5.5	-4.5	-3.5	-2.1	0.7	4.5	9.0	13.7	16.7	19.5	23.0
19.6	-5.5	-4.5	-3.5	-2.1	0.7	4.5	8.9	13.5	16.6	19.3	22.8
19.8	-5.5	-4.5	-3.5	-2.1	0.7	4.4	8.9	13.4	16.4	19.2	22.5
20.0	-5.4	-4.5	-3.4	-2.1	0.7	4.4	8.8	13.3	16.3	19.0	22.3
20.2	-5.4	-4.4	-3.4	-2.1	0.7	4.4	8.7	13.2	16.1	18.8	22.1
20.4	-5.4	-4.4	-3.4	-2.0	0.7	4.3	8.6	13.1	16.0	18.6	21.9
20.6	-5.4	-4.4	-3.4	-2.0	0.7	4.3	8.6	12.9	15.8	18.5	21.7
20.8	-5.3	-4.4	-3.4	-2.0	0.7	4.3	8.5	12.8	15.7	18.3	21.5
21.0	-5.3	-4.3	-3.3	-2.0	0.7	4.2	8.4	12.7	15.5	18.1	21.3
21.2	-5.3	-4.3	-3.3	-2.0	0.7	4.2	8.3	12.6	15.4	17.9	21.0
21.4	-5.2	-4.3	-3.3	-1.9	0.7	4.2	8.3	12.5	15.2	17.7	20.8
21.6	-5.2	-4.2	-3.2	-1.9	0.7	4.1	8.2	12.4	15.1	17.6	20.6

Table F. Age-related reference intervals (RIs) for central (aortic) augmentation index (cAIx) for females (n=462)

Age (years)	1 th	2.5 th	5 th	10 th	25 th	50 th	75 th	90 th	95 th	97.5 th	99 th
5.2	3.5	6.8	9.7	13.3	19.8	27.5	35.8	43.7	48.6	53.0	58.2
5.4	2.9	6.1	9.0	12.6	18.9	26.5	34.6	42.3	47.2	51.5	56.6
5.6	2.4	5.5	8.4	11.8	18.0	25.5	33.5	41.1	45.8	50.1	55.1
5.8	1.9	4.9	7.7	11.1	17.2	24.6	32.4	39.9	44.6	48.8	53.7
6.0	1.4	4.4	7.2	10.5	16.5	23.7	31.4	38.8	43.4	47.5	52.4
6.2	0.9	3.9	6.6	9.9	15.8	22.9	30.5	37.8	42.3	46.3	51.2
6.4	0.5	3.4	6.1	9.3	15.1	22.1	29.6	36.8	41.2	45.2	50.0
6.6	0.2	3.0	5.6	8.8	14.5	21.4	28.8	35.8	40.3	44.2	48.9
6.8	-0.2	2.6	5.2	8.3	13.9	20.7	28.0	35.0	39.3	43.2	47.8
7.0	-0.5	2.2	4.8	7.8	13.4	20.1	27.3	34.1	38.4	42.3	46.8
7.2	-0.9	1.9	4.4	7.4	12.9	19.5	26.6	33.4	37.6	41.4	45.9
7.4	-1.2	1.5	4.0	7.0	12.4	18.9	25.9	32.6	36.8	40.6	45.0
7.6	-1.4	1.2	3.6	6.6	11.9	18.4	25.3	31.9	36.1	39.8	44.2
7.8	-1.7	0.9	3.3	6.2	11.5	17.8	24.7	31.3	35.4	39.0	43.4
8.0	-1.9	0.6	3.0	5.9	11.1	17.4	24.1	30.6	34.7	38.3	42.6
8.2	-2.2	0.4	2.7	5.6	10.7	16.9	23.6	30.0	34.1	37.6	41.9
8.4	-2.4	0.1	2.4	5.2	10.3	16.5	23.1	29.5	33.5	37.0	41.2
8.6	-2.6	-0.1	2.2	5.0	10.0	16.1	22.7	28.9	32.9	36.4	40.6
8.8	-2.8	-0.3	1.9	4.7	9.7	15.7	22.2	28.4	32.3	35.8	40.0
9.0	-2.9	-0.5	1.7	4.4	9.3	15.3	21.8	28.0	31.8	35.3	39.4
9.2	-3.1	-0.7	1.5	4.2	9.1	15.0	21.4	27.5	31.3	34.8	38.9
9.4	-3.3	-0.9	1.3	4.0	8.8	14.7	21.0	27.1	30.9	34.3	38.3
9.6	-3.4	-1.1	1.1	3.7	8.5	14.4	20.6	26.7	30.4	33.8	37.8
9.8	-3.5	-1.2	0.9	3.5	8.3	14.1	20.3	26.3	30.0	33.4	37.4
10.0	-3.7	-1.4	0.7	3.4	8.1	13.8	20.0	25.9	29.6	33.0	36.9
10.2	-3.8	-1.5	0.6	3.2	7.9	13.5	19.7	25.6	29.3	32.6	36.5
10.4	-3.9	-1.6	0.4	3.0	7.7	13.3	19.4	25.3	28.9	32.2	36.1
10.6	-4.0	-1.8	0.3	2.9	7.5	13.1	19.1	24.9	28.6	31.8	35.7
10.8	-4.1	-1.9	0.2	2.7	7.3	12.9	18.9	24.7	28.3	31.5	35.4
11.0	-4.2	-2.0	0.1	2.6	7.1	12.7	18.6	24.4	28.0	31.2	35.0
11.2	-4.3	-2.1	0.0	2.5	7.0	12.5	18.4	24.1	27.7	30.9	34.7
11.4	-4.3	-2.2	-0.1	2.3	6.8	12.3	18.2	23.9	27.4	30.6	34.4
11.6	-4.4	-2.2	-0.2	2.2	6.7	12.1	18.0	23.7	27.2	30.4	34.1
11.8	-4.5	-2.3	-0.3	2.1	6.6	12.0	17.8	23.4	27.0	30.1	33.9
12.0	-4.5	-2.4	-0.4	2.0	6.4	11.8	17.7	23.2	26.7	29.9	33.6
12.2	-4.6	-2.5	-0.5	1.9	6.3	11.7	17.5	23.1	26.5	29.7	33.4
12.4	-4.6	-2.5	-0.5	1.9	6.2	11.6	17.3	22.9	26.3	29.4	33.2
12.6	-4.7	-2.6	-0.6	1.8	6.1	11.5	17.2	22.7	26.2	29.3	32.9
12.8	-4.7	-2.6	-0.7	1.7	6.1	11.3	17.1	22.6	26.0	29.1	32.8
13.0	-4.7	-2.7	-0.7	1.7	6.0	11.3	17.0	22.4	25.9	28.9	32.6
13.2	-4.8	-2.7	-0.8	1.6	5.9	11.2	16.8	22.3	25.7	28.8	32.4
13.4	-4.8	-2.7	-0.8	1.6	5.9	11.1	16.7	22.2	25.6	28.6	32.2
13.6	-4.8	-2.7	-0.8	1.5	5.8	11.0	16.7	22.1	25.5	28.5	32.1
13.8	-4.8	-2.8	-0.9	1.5	5.8	10.9	16.6	22.0	25.3	28.4	32.0
14.0	-4.8	-2.8	-0.9	1.5	5.7	10.9	16.5	21.9	25.2	28.3	31.9
14.2	-4.8	-2.8	-0.9	1.5	5.7	10.8	16.4	21.8	25.2	28.2	31.7
14.4	-4.9	-2.8	-0.9	1.4	5.7	10.8	16.4	21.7	25.1	28.1	31.6
14.6	-4.9	-2.8	-0.9	1.4	5.6	10.8	16.3	21.7	25.0	28.0	31.6
14.8	-4.8	-2.8	-0.9	1.4	5.6	10.7	16.3	21.6	24.9	27.9	31.5
15.0	-4.8	-2.8	-0.9	1.4	5.6	10.7	16.3	21.6	24.9	27.9	31.4
15.2	-4.8	-2.8	-0.9	1.4	5.6	10.7	16.2	21.5	24.8	27.8	31.3
15.4	-4.8	-2.8	-0.9	1.4	5.6	10.7	16.2	21.5	24.8	27.8	31.3
15.6	-4.8	-2.8	-0.9	1.4	5.6	10.7	16.2	21.5	24.8	27.7	31.3
15.8	-4.8	-2.7	-0.9	1.4	5.6	10.7	16.2	21.5	24.8	27.7	31.2
16.0	-4.8	-2.7	-0.8	1.5	5.6	10.7	16.2	21.4	24.7	27.7	31.2
16.2	-4.7	-2.7	-0.8	1.5	5.6	10.7	16.2	21.4	24.7	27.7	31.2
16.4	-4.7	-2.7	-0.8	1.5	5.7	10.7	16.2	21.4	24.7	27.7	31.2
16.6	-4.7	-2.6	-0.8	1.5	5.7	10.7	16.2	21.5	24.7	27.7	31.2
16.8	-4.6	-2.6	-0.7	1.6	5.7	10.8	16.2	21.5	24.8	27.7	31.2
17.0	-4.6	-2.6	-0.7	1.6	5.8	10.8	16.3	21.5	24.8	27.7	31.2
17.2	-4.6	-2.5	-0.6	1.7	5.8	10.8	16.3	21.5	24.8	27.7	31.2
17.4	-4.5	-2.5	-0.6	1.7	5.8	10.9	16.3	21.6	24.8	27.8	31.2
17.6	-4.5	-2.4	-0.6	1.7	5.9	10.9	16.4	21.6	24.9	27.8	31.3
17.8	-4.4	-2.4	-0.5	1.8	5.9	11.0	16.4	21.7	24.9	27.8	31.3
18.0	-4.4	-2.3	-0.5	1.9	6.0	11.0	16.5	21.7	25.0	27.9	31.4
18.2	-4.3	-2.3	-0.4	1.9	6.1	11.1	16.6	21.8	25.0	27.9	31.4
18.4	-4.3	-2.2	-0.3	2.0	6.1	11.2	16.6	21.8	25.1	28.0	31.5
18.6	-4.2	-2.2	-0.3	2.0	6.2	11.2	16.7	21.9	25.2	28.1	31.6
18.8	-4.1	-2.1	-0.2	2.1	6.3	11.3	16.8	22.0	25.2	28.2	31.6
19.0	-4.1	-2.0	-0.1	2.2	6.3	11.4	16.8	22.1	25.3	28.2	31.7
19.2	-4.0	-2.0	-0.1	2.3	6.4	11.5	16.9	22.1	25.4	28.3	31.8
19.4	-3.9	-1.9	0.0	2.3	6.5	11.6	17.0	22.2	25.5	28.4	31.9
19.6	-3.9	-1.8	0.1	2.4	6.6	11.7	17.1	22.3	25.6	28.5	32.0
19.8	-3.8	-1.7	0.2	2.5	6.7	11.7	17.2	22.4	25.7	28.6	32.1
20.0	-3.7	-1.7	0.3	2.6	6.8	11.8	17.3	22.5	25.8	28.7	32.2
20.2	-3.7	-1.6	0.3	2.7	6.9	11.9	17.4	22.6	25.9	28.8	32.3
20.4	-3.6	-1.5	0.4	2.8	7.0	12.0	17.5	22.8	26.0	29.0	32.4
20.6	-3.5	-1.4	0.5	2.9	7.1	12.2	17.6	22.9	26.2	29.1	32.6
20.8	-3.4	-1.3	0.6	3.0	7.2	12.3	17.8	23.0	26.3	29.2	32.7
21.0	-3.3	-1.2	0.7	3.1	7.3	12.4	17.9	23.1	26.4	29.3	32.8
21.2	-3.2	-1.1	0.8	3.2	7.4	12.5	18.0	23.3	26.5	29.5	33.0
21.4	-3.2	-1.0	0.9	3.3	7.5	12.6	18.1	23.4	26.7	29.6	33.1
21.6	-3.1	-0.9	1.0	3.4	7.6	12.8	18.3	23.5	26.8	29.8	33.3
21.8	-3.0	-0.8	1.1	3.5	7.7	12.9	18.4	23.7	27.0	29.9	33.4

Table G. Body height-related reference intervals (RIs) for central (aortic) augmentation index (cAIx) for males (n=576)

Body Height (cm)	1 th	2.5 th	5 th	10 th	25 th	50 th	75 th	90 th	95 th	97.5 th	99 th
104	5.06	6.36	7.54	8.95	11.43	14.41	17.59	20.62	22.51	24.20	26.23
105	4.79	6.12	7.32	8.76	11.31	14.35	17.62	20.74	22.69	24.44	26.52
106	4.53	5.89	7.10	8.57	11.18	14.30	17.65	20.86	22.86	24.66	26.81
107	4.28	5.65	6.89	8.39	11.04	14.24	17.67	20.96	23.02	24.87	27.08
108	4.03	5.42	6.68	8.20	10.91	14.17	17.69	21.06	23.18	25.07	27.34
109	3.78	5.19	6.47	8.02	10.77	14.10	17.70	21.15	23.32	25.26	27.59
110	3.54	4.97	6.26	7.83	10.64	14.03	17.70	21.23	23.45	25.44	27.83
111	3.30	4.74	6.05	7.65	10.50	13.95	17.70	21.30	23.57	25.61	28.06
112	3.06	4.52	5.85	7.46	10.36	13.87	17.69	21.37	23.69	25.77	28.27
113	2.83	4.30	5.65	7.28	10.21	13.79	17.67	21.42	23.79	25.92	28.47
114	2.60	4.09	5.44	7.10	10.07	13.70	17.65	21.47	23.89	26.06	28.67
115	2.38	3.87	5.24	6.91	9.93	13.61	17.63	21.52	23.98	26.19	28.85
116	2.16	3.66	5.04	6.73	9.78	13.51	17.59	21.55	24.05	26.31	29.02
117	1.94	3.46	4.85	6.55	9.63	13.41	17.56	21.58	24.12	26.42	29.18
118	1.73	3.25	4.65	6.37	9.48	13.31	17.51	21.60	24.19	26.52	29.33
119	1.52	3.05	4.46	6.19	9.33	13.21	17.47	21.61	24.24	26.61	29.46
120	1.31	2.85	4.26	6.01	9.18	13.10	17.41	21.61	24.28	26.69	29.59
121	1.11	2.65	4.07	5.83	9.03	12.99	17.35	21.61	24.32	26.76	29.71
122	0.91	2.45	3.88	5.65	8.88	12.87	17.29	21.61	24.35	26.83	29.82
123	0.71	2.26	3.70	5.47	8.72	12.76	17.22	21.59	24.37	26.88	29.91
124	0.52	2.07	3.51	5.30	8.57	12.64	17.15	21.57	24.38	26.93	30.00
125	0.33	1.88	3.33	5.12	8.41	12.52	17.07	21.54	24.39	26.96	30.08
126	0.14	1.69	3.14	4.94	8.26	12.39	16.99	21.51	24.39	26.99	30.14
127	-0.05	1.51	2.96	4.77	8.10	12.27	16.91	21.47	24.38	27.01	30.20
128	-0.23	1.33	2.78	4.60	7.94	12.14	16.82	21.42	24.36	27.03	30.25
129	-0.41	1.15	2.60	4.42	7.78	12.01	16.72	21.37	24.34	27.03	30.29
130	-0.58	0.97	2.43	4.25	7.62	11.87	16.62	21.31	24.31	27.03	30.32
131	-0.75	0.79	2.25	4.08	7.46	11.73	16.52	21.25	24.27	27.01	30.34
132	-0.92	0.62	2.08	3.91	7.30	11.60	16.41	21.18	24.23	27.00	30.35
133	-1.09	0.45	1.91	3.74	7.14	11.46	16.30	21.10	24.18	26.97	30.35
134	-1.25	0.28	1.74	3.57	6.98	11.31	16.19	21.02	24.12	26.93	30.35
135	-1.41	0.11	1.57	3.40	6.82	11.17	16.07	20.93	24.06	26.89	30.33
136	-1.57	-0.05	1.40	3.23	6.66	11.02	15.95	20.84	23.99	26.84	30.31
137	-1.73	-0.21	1.23	3.06	6.50	10.87	15.82	20.75	23.91	26.79	30.28
138	-1.88	-0.37	1.07	2.90	6.33	10.72	15.70	20.64	23.83	26.72	30.24
139	-2.03	-0.53	0.91	2.73	6.17	10.57	15.56	20.54	23.74	26.65	30.19
140	-2.18	-0.69	0.75	2.57	6.01	10.42	15.43	20.43	23.65	26.58	30.14
141	-2.32	-0.84	0.59	2.41	5.84	10.26	15.29	20.31	23.55	26.49	30.08
142	-2.47	-0.99	0.43	2.24	5.68	10.11	15.15	20.19	23.44	26.40	30.01
143	-2.61	-1.14	0.27	2.08	5.52	9.95	15.00	20.06	23.33	26.31	29.93
144	-2.74	-1.29	0.12	1.92	5.35	9.79	14.86	19.93	23.21	26.20	29.84
145	-2.88	-1.44	-0.03	1.76	5.19	9.63	14.71	19.80	23.09	26.09	29.75
146	-3.01	-1.58	-0.19	1.61	5.03	9.47	14.55	19.66	22.97	25.98	29.65
147	-3.14	-1.72	-0.33	1.45	4.86	9.30	14.40	19.52	22.83	25.86	29.54
148	-3.27	-1.86	-0.48	1.29	4.70	9.14	14.24	19.37	22.70	25.73	29.43
149	-3.39	-2.00	-0.63	1.14	4.54	8.97	14.08	19.22	22.55	25.60	29.31
150	-3.51	-2.13	-0.77	0.99	4.37	8.81	13.91	19.07	22.41	25.46	29.18
151	-3.63	-2.27	-0.92	0.83	4.21	8.64	13.75	18.91	22.26	25.32	29.05
152	-3.75	-2.40	-1.06	0.68	4.05	8.47	13.58	18.75	22.10	25.17	28.91
153	-3.87	-2.53	-1.20	0.53	3.89	8.30	13.41	18.58	21.94	25.01	28.77
154	-3.98	-2.66	-1.34	0.38	3.72	8.13	13.24	18.41	21.77	24.85	28.61
155	-4.09	-2.78	-1.48	0.23	3.56	7.96	13.06	18.24	21.61	24.69	28.46
156	-4.20	-2.91	-1.61	0.09	3.40	7.78	12.89	18.06	21.43	24.52	28.29
157	-4.30	-3.03	-1.74	-0.06	3.24	7.61	12.71	17.88	21.26	24.34	28.12
158	-4.41	-3.15	-1.88	-0.20	3.08	7.44	12.53	17.70	21.07	24.16	27.95
159	-4.51	-3.27	-2.01	-0.35	2.92	7.26	12.34	17.51	20.89	23.98	27.77
160	-4.61	-3.38	-2.14	-0.49	2.76	7.09	12.16	17.33	20.70	23.79	27.58
161	-4.71	-3.50	-2.26	-0.63	2.60	6.91	11.97	17.14	20.51	23.60	27.39
162	-4.80	-3.61	-2.39	-0.77	2.44	6.74	11.79	16.94	20.31	23.40	27.19
163	-4.89	-3.72	-2.51	-0.91	2.28	6.56	11.60	16.74	20.11	23.20	26.99
164	-4.99	-3.83	-2.64	-1.04	2.12	6.38	11.41	16.54	19.91	23.00	26.78
165	-5.07	-3.93	-2.76	-1.18	1.96	6.21	11.21	16.34	19.70	22.79	26.57
166	-5.16	-4.04	-2.88	-1.31	1.81	6.03	11.02	16.14	19.49	22.57	26.35
167	-5.24	-4.14	-2.99	-1.45	1.65	5.85	10.83	15.93	19.28	22.36	26.13
168	-5.33	-4.24	-3.11	-1.58	1.50	5.68	10.63	15.72	19.06	22.14	25.91
169	-5.41	-4.34	-3.22	-1.71	1.34	5.50	10.43	15.51	18.84	21.91	25.68
170	-5.49	-4.44	-3.34	-1.84	1.19	5.32	10.23	15.30	18.62	21.68	25.44
171	-5.56	-4.54	-3.45	-1.97	1.03	5.14	10.03	15.08	18.40	21.45	25.21
172	-5.64	-4.63	-3.56	-2.09	0.88	4.96	9.83	14.86	18.17	21.22	24.96
173	-5.71	-4.72	-3.67	-2.22	0.73	4.78	9.63	14.64	17.94	20.98	24.72
174	-5.78	-4.81	-3.77	-2.34	0.58	4.61	9.43	14.42	17.71	20.74	24.47
175	-5.85	-4.90	-3.88	-2.47	0.43	4.43	9.23	14.20	17.47	20.50	24.21
176	-5.91	-4.99	-3.98	-2.59	0.28	4.25	9.02	13.97	17.24	20.25	23.96
177	-5.98	-5.07	-4.08	-2.71	0.13	4.07	8.82	13.75	17.00	20.00	23.70
178	-6.04	-5.15	-4.18	-2.83	-0.02	3.90	8.61	13.52	16.76	19.75	23.43
179	-6.10	-5.23	-4.28	-2.94	-0.16	3.72	8.41	13.29	16.51	19.49	23.16
180	-6.16	-5.31	-4.38	-3.06	-0.31	3.54	8.20	13.06	16.27	19.24	22.89
181	-6.21	-5.39	-4.47	-3.17	-0.45	3.36	8.00	12.82	16.02	18.98	22.62
182	-6.27	-5.47	-4.56	-3.28	-0.60	3.19	7.79	12.59	15.77	18.71	22.34
183	-6.32	-5.54	-4.66	-3.40	-0.74	3.01	7.58	12.36	15.52	18.45	22.06
184	-6.37	-5.61	-4.75	-3.51	-0.88	2.84	7.37	12.12	15.27	18.18	21.78
185	-6.42	-5.68	-4.83	-3.61	-1.02	2.66	7.16	11.88	15.02	17.91	21.50
186	-6.46	-5.75	-4.92	-3.72	-1.16	2.49	6.96	11.64	14.76	17.64	21.21
187	-6.51	-5.82	-5.00	-3.83	-1.30	2.31	6.75	11.41	14.50	17.37	20.92
188	-6.55	-5.88	-5.09	-3.93	-1.44	2.14	6.54	11.17	14.24	17.10	20.62
189	-6.59	-5.95	-5.17	-4.03	-1.57	1.97	6.33	10.93	13.98	16.82	20.33
190	-6.63	-6.01	-5.25	-4.13	-1.71	1.80	6.12	10.68	13.72	16.54	20.03
191	-6.67	-6.07	-5.33	-4.23	-1.84	1.62	5.91	10.44	13.46	16.26	19.73

Table H. Body height-related reference intervals (RIs) for central (aortic) augmentation index (cAIx) for females (n=462)

Body Height (cm)	1 th	2.5 th	5 th	10 th	25 th	50 th	75 th	90 th	95 th	97.5 th	99 th
102	-1.63	1.21	3.82	7.03	12.82	19.86	27.47	34.76	39.32	43.40	48.27
103	-1.43	1.39	3.98	7.17	12.89	19.85	27.38	34.57	39.08	43.10	47.91
104	-1.25	1.55	4.13	7.29	12.96	19.85	27.28	34.39	38.84	42.81	47.55
105	-1.07	1.71	4.27	7.40	13.02	19.83	27.18	34.20	38.60	42.52	47.20
106	-0.91	1.86	4.40	7.50	13.06	19.81	27.08	34.02	38.36	42.23	46.85
107	-0.75	1.99	4.51	7.59	13.10	19.78	26.97	33.83	38.12	41.94	46.51
108	-0.61	2.12	4.62	7.67	13.13	19.74	26.85	33.64	37.88	41.66	46.17
109	-0.48	2.23	4.72	7.74	13.16	19.70	26.73	33.44	37.64	41.38	45.84
110	-0.36	2.34	4.80	7.80	13.17	19.65	26.61	33.25	37.40	41.09	45.50
111	-0.24	2.43	4.88	7.86	13.18	19.59	26.49	33.06	37.16	40.81	45.18
112	-0.14	2.52	4.95	7.90	13.17	19.53	26.36	32.86	36.92	40.54	44.85
113	-0.05	2.59	5.00	7.93	13.16	19.46	26.22	32.66	36.68	40.26	44.53
114	0.03	2.66	5.05	7.96	13.15	19.39	26.09	32.46	36.44	39.98	44.21
115	0.10	2.71	5.09	7.98	13.12	19.31	25.95	32.26	36.20	39.71	43.89
116	0.17	2.76	5.12	7.98	13.09	19.22	25.80	32.06	35.96	39.44	43.58
117	0.22	2.79	5.14	7.98	13.05	19.13	25.66	31.86	35.72	39.17	43.27
118	0.26	2.82	5.15	7.97	13.00	19.04	25.50	31.65	35.49	38.90	42.97
119	0.30	2.84	5.15	7.96	12.95	18.94	25.35	31.45	35.25	38.63	42.66
120	0.32	2.85	5.15	7.93	12.88	18.83	25.19	31.24	35.01	38.37	42.36
121	0.34	2.85	5.13	7.90	12.82	18.72	25.03	31.03	34.77	38.10	42.07
122	0.34	2.84	5.11	7.86	12.74	18.60	24.87	30.82	34.53	37.84	41.77
123	0.34	2.82	5.08	7.81	12.66	18.48	24.70	30.61	34.30	37.58	41.48
124	0.33	2.80	5.04	7.75	12.57	18.35	24.53	30.40	34.06	37.31	41.19
125	0.31	2.76	4.99	7.69	12.48	18.22	24.36	30.19	33.82	37.06	40.91
126	0.28	2.72	4.94	7.62	12.38	18.08	24.19	29.98	33.59	36.80	40.62
127	0.25	2.67	4.87	7.54	12.27	17.94	24.01	29.76	33.35	36.54	40.34
128	0.20	2.61	4.80	7.45	12.16	17.80	23.83	29.55	33.11	36.29	40.07
129	0.15	2.55	4.73	7.36	12.04	17.65	23.64	29.33	32.88	36.03	39.79
130	0.09	2.48	4.64	7.26	11.92	17.50	23.46	29.12	32.64	35.78	39.52
131	0.02	2.40	4.55	7.16	11.79	17.34	23.27	28.90	32.41	35.53	39.25
132	-0.05	2.31	4.45	7.05	11.66	17.18	23.08	28.68	32.17	35.28	38.98
133	-0.13	2.22	4.35	6.93	11.52	17.01	22.89	28.46	31.94	35.03	38.71
134	-0.22	2.11	4.24	6.81	11.37	16.84	22.69	28.24	31.70	34.78	38.45
135	-0.32	2.01	4.12	6.68	11.22	16.67	22.49	28.02	31.47	34.53	38.19
136	-0.42	1.89	4.00	6.54	11.07	16.49	22.29	27.80	31.23	34.29	37.93
137	-0.53	1.77	3.87	6.40	10.91	16.31	22.09	27.58	31.00	34.04	37.67
138	-0.65	1.65	3.73	6.26	10.74	16.13	21.89	27.36	30.77	33.80	37.42
139	-0.77	1.51	3.59	6.10	10.57	15.94	21.68	27.13	30.53	33.56	37.16
140	-0.90	1.37	3.44	5.95	10.40	15.75	21.47	26.91	30.30	33.32	36.91
141	-1.03	1.23	3.29	5.78	10.22	15.55	21.26	26.69	30.07	33.08	36.67
142	-1.17	1.08	3.13	5.62	10.04	15.36	21.05	26.46	29.84	32.84	36.42
143	-1.32	0.92	2.97	5.44	9.86	15.16	20.84	26.24	29.60	32.60	36.18
144	-1.47	0.76	2.80	5.27	9.67	14.95	20.62	26.01	29.37	32.37	35.93
145	-1.63	0.60	2.62	5.09	9.47	14.75	20.40	25.78	29.14	32.13	35.69
146	-1.79	0.43	2.45	4.90	9.27	14.54	20.18	25.56	28.91	31.90	35.46
147	-1.96	0.25	2.26	4.71	9.07	14.33	19.96	25.33	28.68	31.66	35.22
148	-2.13	0.07	2.08	4.51	8.87	14.11	19.74	25.10	28.45	31.43	34.99
149	-2.30	-0.12	1.88	4.31	8.66	13.89	19.52	24.87	28.22	31.20	34.75
150	-2.48	-0.30	1.69	4.11	8.45	13.67	19.29	24.64	27.99	30.97	34.52
151	-2.67	-0.50	1.49	3.91	8.23	13.45	19.06	24.41	27.76	30.74	34.29
152	-2.85	-0.69	1.28	3.70	8.01	13.23	18.84	24.19	27.53	30.51	34.07
153	-3.05	-0.90	1.08	3.48	7.79	13.00	18.61	23.96	27.30	30.28	33.84
154	-3.24	-1.10	0.86	3.26	7.56	12.77	18.37	23.73	27.07	30.06	33.62
155	-3.44	-1.31	0.65	3.04	7.34	12.54	18.14	23.49	26.84	29.83	33.40
156	-3.64	-1.52	0.43	2.82	7.11	12.30	17.91	23.26	26.62	29.61	33.18
157	-3.85	-1.73	0.21	2.59	6.87	12.07	17.67	23.03	26.39	29.38	32.96
158	-4.06	-1.95	-0.01	2.36	6.64	11.83	17.44	22.80	26.16	29.16	32.74
159	-4.27	-2.17	-0.24	2.13	6.40	11.59	17.20	22.57	25.93	28.94	32.53
160	-4.48	-2.40	-0.47	1.89	6.16	11.35	16.96	22.34	25.71	28.72	32.31
161	-4.69	-2.62	-0.70	1.65	5.91	11.10	16.72	22.11	25.48	28.50	32.10
162	-4.91	-2.85	-0.94	1.41	5.67	10.86	16.48	21.87	25.26	28.28	31.89
163	-5.13	-3.08	-1.18	1.17	5.42	10.61	16.24	21.64	25.03	28.06	31.68
164	-5.35	-3.31	-1.41	0.93	5.17	10.36	16.00	21.41	24.81	27.84	31.47
165	-5.57	-3.55	-1.66	0.68	4.92	10.11	15.75	21.18	24.58	27.63	31.27
166	-5.80	-3.78	-1.90	0.43	4.66	9.86	15.51	20.94	24.36	27.41	31.06
167	-6.02	-4.02	-2.14	0.18	4.41	9.60	15.26	20.71	24.13	27.20	30.86
168	-6.25	-4.26	-2.39	-0.07	4.15	9.35	15.02	20.48	23.91	26.98	30.66
169	-6.47	-4.50	-2.64	-0.33	3.89	9.09	14.77	20.24	23.69	26.77	30.46
170	-6.70	-4.74	-2.89	-0.58	3.63	8.83	14.52	20.01	23.46	26.56	30.26
171	-6.93	-4.98	-3.14	-0.84	3.37	8.58	14.27	19.78	23.24	26.35	30.06
172	-7.15	-5.22	-3.39	-1.10	3.11	8.32	14.03	19.54	23.02	26.13	29.87
173	-7.38	-5.46	-3.64	-1.36	2.84	8.05	13.78	19.31	22.80	25.92	29.67
174	-7.60	-5.70	-3.89	-1.62	2.58	7.79	13.53	19.08	22.58	25.72	29.48
175	-7.83	-5.95	-4.15	-1.88	2.31	7.53	13.28	18.84	22.36	25.51	29.29
176	-8.05	-6.19	-4.40	-2.14	2.04	7.27	13.02	18.61	22.14	25.30	29.10
177	-8.27	-6.43	-4.65	-2.40	1.77	7.00	12.77	18.38	21.92	25.09	28.91
178	-8.49	-6.67	-4.90	-2.67	1.50	6.74	12.52	18.14	21.70	24.89	28.72
